# *prominin-1*-null *Xenopus laevis* develop subretinal drusenoid-like deposits, cone-rod dystrophy and RPE atrophy

**DOI:** 10.1242/jcs.262298

**Published:** 2024-11-12

**Authors:** Brittany J. Carr, Dominic Skitsko, Linnea M. Kriese, Jun Song, Zixuan Li, Myeong Jin Ju, Orson L. Moritz

**Affiliations:** ^1^ The University of Alberta, Faculty of Medicine and Dentistry, Department of Ophthalmology and Visual Sciences, Edmonton, AB T6G 2E1, Canada; ^2^ The University of Alberta, Faculty of Medicine and Dentistry, Department of Cell Biology, Edmonton, AB T6G 2H7, Canada; ^3^ The University of British Columbia, Faculty of Medicine, Department of Ophthalmology and Visual Sciences, Vancouver, BC V5Z 0A6, Canada; ^4^ The University of British Columbia, Faculty of Applied Science, Faculty of Medicine, School of Biomedical Engineering, Vancouver, BC V6T 2B9, Canada

**Keywords:** Outer segment, Prominin-1, Retinal degeneration, Retinal pigment epithelium, Subretinal drusenoid deposit, *Xenopus laevis*

## Abstract

Prominin-1 (*PROM1*) variants are associated with inherited, non-syndromic vision loss. We used CRISPR/Cas9 to induce *prom1*-null mutations in *Xenopus laevis* and then tracked retinal disease progression from the ages of 6 weeks to 3 years. We found that *prom1*-null-associated retinal degeneration in frogs was age-dependent and involved retinal pigment epithelium (RPE) dysfunction preceding photoreceptor degeneration. Before photoreceptor degeneration occurred, aging *prom1*-null frogs developed larger and increasing numbers of cellular debris deposits in the subretinal space and outer segment layer, which resembled subretinal drusenoid deposits (SDDs) in their location, histology and representation as seen by color fundus photography and optical coherence tomography (OCT). Evidence for an RPE origin of these deposits included infiltration of pigment granules into the deposits, thinning of the RPE as measured by OCT, and RPE disorganization as measured by histology and OCT. The appearance and accumulation of SDD-like deposits and RPE thinning and disorganization in our animal model suggests an underlying disease mechanism for *prom1*-null-mediated blindness that involves death and dysfunction of the RPE preceding photoreceptor degeneration, instead of direct effects upon photoreceptor outer segment morphogenesis, as was previously hypothesized.

## Teaser

**Summary:** *PROM1* variants are associated with inherited blindness. CRISPR knockdown of *prom1* in frogs results in age-dependent retinal degeneration that involves retinal pigment epithelium dysfunction preceding photoreceptor death.

## INTRODUCTION

Inherited retinal degeneration is a heterogeneous set of disorders caused by variants in genes important for retinal function that result in progressive vision loss and blindness. Advances in genetic screening have identified hundreds of genes and variants potentially linked to inherited retinal degeneration. However, relatively little is known about the functions of these genes, genotype–phenotype correlation for variants, or the underlying mechanisms by which variants cause blindness. These disorders include retinal disorders linked to variants in prominin-1 (*PROM1*). *PROM1*-null variants are associated with autosomal recessive cone-rod dystrophy, retinitis pigmentosa and macular degeneration (Maw et al., 2000; Permanyer et al., 2010; Pras et al., 2009; Zhang et al., 2007). The allele with the point variant *PROM^R373C^* is linked to autosomal dominant Stargardt-like juvenile macular degeneration (Michaelides et al., 2010). Mouse and zebrafish studies concluded that retinal degeneration caused by *prom1* mutations was caused by defective outer segment disc morphogenesis (Lu et al., 2019; Yang et al., 2008; Zacchigna et al., 2009).

Modelling *prom1*-null mutations in *Xenopus laevis*, however, has shown that although photoreceptor outer segments are dysmorphic, outer segment morphogenesis and visual function is maintained over time (Carr et al., 2021). *X. laevis* are long-lived (15–20 years) diurnal animals with retinas comprising ∼47% cones. They do not have a macula or visual streak; instead, their photoreceptors are expressed in a uniform mosaic pattern across the entirety of the retina (Wilhelm and Gábriel, 1999). Regardless, they are a useful animal for the study of cones and cone-related disease because they have large cone outer segments, they express similar cone opsins to those in humans (Röhlich and Szél, 2000), and they have a longstanding history of studies of photoreceptor development, retinal development and retinal circuitry (Witkovsky, 2000). Here, we report that with age (≥6 months), autofluorescent deposits appear in the outer segment layer between the inner segment/outer segment boundary and the retinal pigment epithelium (RPE) in *prom1*-null *X. laevis*. These deposits increase in size and number as animals continue aging and resemble human subretinal drusenoid deposits (SDDs) (Zweifel et al., 2010).

Human SDDs are also termed reticular pseudodrusen and differ from conventional soft and hard drusen in their localization and composition (Wightman and Guymer, 2019). SDDs localize to the subretinal space, between the retinal outer segments and the apical side of the RPE. Soft drusen are present external to the basal border of the RPE, between the RPE and Bruch's membrane (Curcio, 2018). Oil Red O, a neutral lipid stain, colors soft drusen a vivid red but does not stain SDDs, suggesting different lipid compositions and sources (Greferath et al., 2016; Oak et al., 2014). Human SDDs contain unesterified cholesterol, apolipoprotein E (apoE), complement factor H and vitronectin, whereas soft drusen contain apoB, apoA-1 and esterified cholesterol (Greferath et al., 2016; Rudolf et al., 2008; Sarks et al., 1988; Wightman and Guymer, 2019). However, the full protein and lipid composition of SDDs is not known and there is controversy as to the presence of proteins such as rod and cone opsins, which would indicate outer segment membrane components in SDDs (Greferath et al., 2016; Rudolf et al., 2008).

The prevalence of SDDs and their role in retinal degenerative disease has historically been unknown. This is due in part to the difficulty in detecting SDDs with color fundus photography coupled with the lack of an animal model (Fletcher et al., 2014; Zarubina et al., 2016). There is mounting evidence that SDDs are present in the retinas of most people, irrespective of whether they have retinal degenerative disease (Zarubina et al., 2016). However, at some point, larger or increased numbers of SDDs tip the scales from a benign occurrence to a pathogenic trigger, increasing the risk for severe retinal degenerative disease, especially for dry (geographic atrophy) and wet (choroidal neovascularization, CNV) age-related macular degeneration (AMD) (Wightman and Guymer, 2019). The cause of this tipping point remains elusive.

Here, we demonstrate that aging *prom1*-null *X. laevis* develop deposits of cellular debris that resemble SDDs. Affected frogs showed analogous effects to severe cone-rod retinal degenerative disease and some hallmarks of dry AMD such as localized retinal atrophy, RPE migration and/or death, and the absence of CNV. These findings call into question previous conclusions that disrupted outer segment morphogenesis is the direct cause of *prom1*-null-associated retinal degeneration (Carr et al., 2021; Lu et al., 2019; Zacchigna et al., 2009). Rather, it appears that secondary toxic effects, such as accumulation of these SDD-like deposits or dysfunction or death of RPE that occurs with age, might be instead responsible for photoreceptor death caused by *prom1*-null mutations. Thus, these data support a potential novel target therapeutic pathway for *prom1*-null mutations: secondary toxic effects on the RPE. They also establish *X. laevis* as a potential animal model for the investigation and therapeutic development of some of the key hallmarks of dry AMD.

## RESULTS

**Fig. 1.**
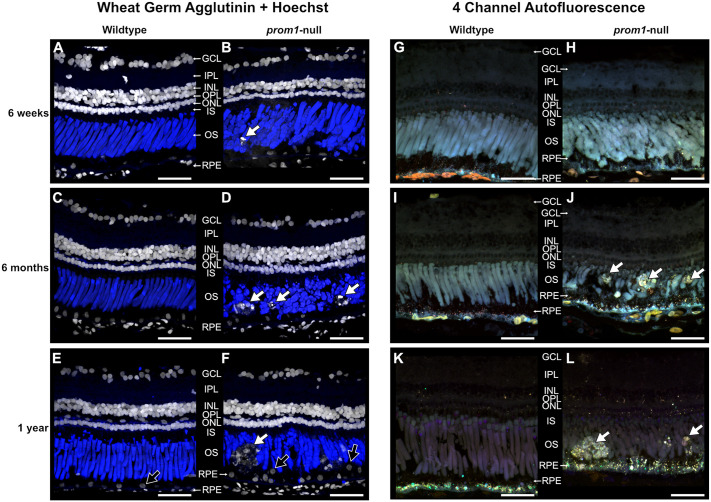
***prom1*-null frogs develop subretinal deposits beginning at 6 months of age.** Representative histology of wildtype and F0 *prom1*-null retinas in animals aged 6 weeks to 1 year examined with Hoechst (white) and wheat germ agglutinin (blue) labeling (A–F) and four-channel label-less autofluorescence combining 405, 488, 555 and 647 nm excitation wavelengths (G–L). Autofluorescence images are color coded to correspond to the detected emission spectra: cyan (405 nm), green (488 nm), orange (555 nm) and red (647 nm). Between 6 weeks and 1 year of age, deposits of cellular debris in the outer segment layer accumulated and increased significantly in size (white arrows). These deposits were strongly labelled with the Hoechst dye (white; A–F) and were not present in wildtype control animals. Hoechst staining also demonstrates the progressive disorganization of retinal pigment epithelium (RPE) nuclei (F, black arrows). When examined using autofluorescence (G–L), the deposits fluoresced strongly at different excitation spectra than those of the surrounding outer segment material, and there was an increase in the number of autofluorescent puncta in the RPE layer of *prom1*-null frogs (H,J,L) compared to that in the wildtype controls (G,I,K). Scale bars: 50 µm. Abbreviations: GCL, ganglion cell layer; INL, inner nuclear layer; IPL, inner plexiform layer; IS, inner segments; ONL, outer nuclear layer; OPL, outer plexiform layer; OS, outer segments. Number of animals: *n*=10 (A), 13 (B), 5 (C,D,J), 3 (E,I,K), 6 (F,L), 4 (G,H).

In *prom1*-null *X. laevis*, loss of the Prom1 protein resulted in dysmorphic rod and cone outer segments (Fig. 1; Fig. S1). Rod outer segments comprised large overgrown whorls of membranes within the plasma membrane, whereas the cone outer segments were elongated and fragmented, with cone opsin-positive material dotting the outside of rod outer segments. Although the photoreceptors were severely dysmorphic, *prom1*-null retinas did not degenerate quickly. Instead, small puncta that labeled heavily with the Hoechst 33342 (hereafter referred to as Hoechst) nuclear stain appeared in the outer segment layer by 6 weeks (Fig. 1A–F). By 6 months, these puncta coalesced into larger deposits of cellular debris that resembled human SDDs in localization, and by 1 year, very large deposits (35–60 µm) were present (Fig. 1). These large SDD-like deposits autofluoresced at wavelengths distinct from those of the surrounding outer segment material when viewed with label-less four-channel confocal microscopy, and there was a concurrent increase in lipofuscin-like autofluorescence in the RPE of *prom1*-null animals compared to that in wildtype animals (Fig. 1G–L). In addition to the presence of large SDD-like deposits in the outer segment layer, organizational defects in the RPE were visible by displaced nuclei (Fig. 1F, black arrows) and increased RPE autofluorescence in *prom1*-null mutants at one year of age (Fig. 1L). Interestingly, we observed during our investigations that Prom1 immunoreactivity decreased in the central retina of wildtype animals with full-length rod outer segments [42 days post fertilization (dpf)] compared to that in much younger animals, which had outer segments that were actively elongating in addition to maintaining daily disc synthesis (14 dpf) (Fig. S1). Prom1 immunoreactivity remained strong in the periphery of adult eyes, where outer segments were younger and actively elongating.

**Fig. 2.**
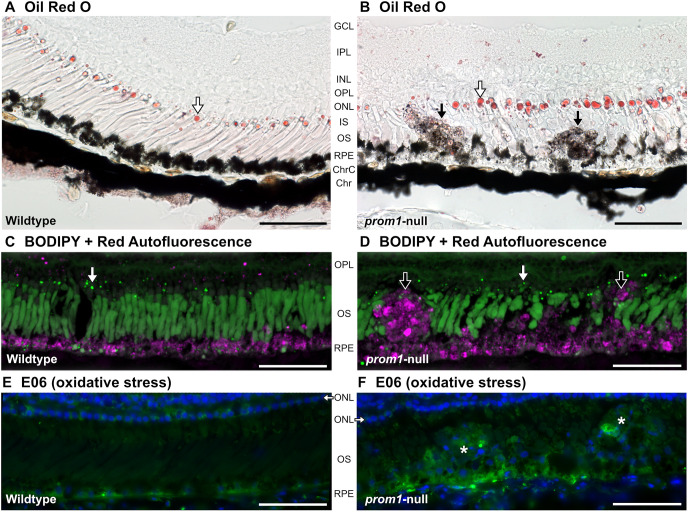
**Retinal deposits associated with *prom1*-null mutants are negative for neutral lipids, but contain oxidized phospholipids.** Representative images of Oil Red O, BODIPY and E06 labeling in 1- to 3-year-old wildtype (A,C,E) and *prom1*-null (B,D,F) retinas. Both Oil Red O (A,B) and BODIPY (C,D) labeled the oil droplets in the cone photoreceptors (white arrows), but neither labeled the subretinal drusen deposit (SDD)-like deposits in the *prom1*-null retinas strongly (black arrows). Red autofluorescence was detected in the RPE of wildtype frogs and in the RPE and large deposits in *prom1*-null frogs (C,D; magenta). E06 labeled the RPE moderately in wildtype frogs (E). In *prom1*-null frogs, the RPE was labeled more strongly, and it looked similar to red autofluorescence (D) in that it penetrated more deeply into the outer segment layer. The deposits were labeled heterogeneously with E06, and they were more immunopositive than the surrounding outer segments, where no deposits appeared (asterisks, F). The RPE was disrupted in *prom1*-null retinas; pigment granules infiltrated into the SDD-like deposits (B,F) and migrated further into the outer segment layer than in wildtype animals (B,D,F). Scale bars: 50 µm. Number of animals: wildtype, *n*=5; *prom1*-null, *n*=9. Chr, choroid; ChrC, choriocapillaris; GCL, ganglion cell layer; INL, inner nuclear layer; IPL, inner plexiform layer; IS, inner segments; ONL, outer nuclear layer; OPL, outer plexiform layer; OS, outer segments; RPE, retinal pigment epithelium.

To investigate further similarities between these *X. laevis* SDD-like deposits and human SDDs, labeling was performed with Oil Red O and BODIPY, which labels neural lipids but does not label human SDDs. Our frog SDD-like deposits mimicked labeled results in human SDDs, and did not label strongly with Oil Red O or BODIPY (Fig. 2A–D), indicating a lack of neutral lipids such as cholesterol esters and triacylglycerol (Bharati et al., 2022; Elle et al., 2010; Ramírez-Zacarías et al., 1992). Brightfield and red autofluorescence imaging also revealed that the RPE in *prom1*-null animals was fragmented, extended much further into the outer segment layer, and infiltrated into the SDD-like deposits compared to that in wildtype animals (Fig. 2A,B). To test for indicators of oxidative stress, we used the E06 antibody to label oxidized phospholipids (Palinski et al., 1996). In wildtype animals, there was very little immunopositive signal for E06 in the outer segments, but there was increased labeling in the RPE (Fig. 2E). SDD-like deposits in *prom1*-null animals demonstrated moderately strong immunoreactivity that was heterogeneously dispersed within the deposits (Fig. 2F). Increased E06 labeling was also observed in the putative RPE that infiltrated deeper into the outer segment layer, similar to the red autofluorescence and RPE pigment granule infiltration seen in Fig. 2B,D.

**Fig. 3.**
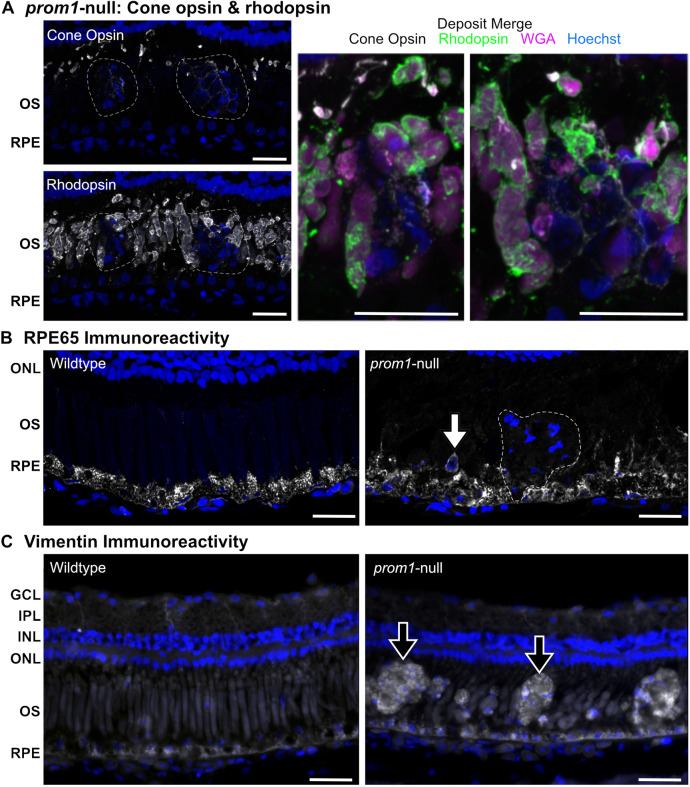
**SDD-like deposits do not contain rhodopsin, cone opsin or RPE65, and are immunopositive for vimentin.** Labeling of SDD-like deposits with markers for (A) outer segments, (B) the retinal pigment epithelium (RPE) and (C) vimentin. SDD-like deposits did not show appreciable fluorescence signal for the outer segment markers wheat germ agglutinin (WGA), rhodopsin or cone opsin (A, dashed regions, merge). Cone opsin was not detected in the deposits, but the cone opsin-positive material surrounded the deposits in a honeycomb-like pattern (A, cone opsin, merge). Large deposits containing multiple nuclei were negative for RPE65 (B, dashed region), but single nuclei displaced from the RPE into the outer segment layer were surrounded by a ring of strong RPE65-positive immunoreactivity (B; white arrow). Vimentin-positive immunoreactivity was increased in the RPE of *prom1*-null animals as well as in the SDD-like deposits (black arrows) but stayed relatively consistent in the cytoskeleton of the Müller glia in the inner plexiform layer (C). Scale bars: 25 µm. GCL, ganglion cell layer; INL, inner nuclear layer; IPL, inner plexiform layer; ONL, outer nuclear layer; OS, outer segments. Numbers of animals: wildtype, *n*=3–6; *prom1*-null, *n*=3–5.

The SDD-like deposits in *prom1*-null animals did not contain significant amounts of rhodopsin- or wheat germ agglutinin (WGA)-positive labeling, but they did contain a small amount of cone opsin-positive material that showed up as a honeycomb-like shape within the deposits (Fig. 3A). Large deposits made up of multiple labeled nuclei did not contain RPE65 labeling, but some single nuclei displaced into the outer segment layer were surrounded by a ring of strong RPE65-positive immunoreactivity (Fig. 3B). The relative level of vimentin-positive signal was higher in the SDD-like deposits and in the RPE surrounding these deposits in *prom1*-null animals compared to that in wildtype counterparts (Fig. 3C).

**Fig. 4.**
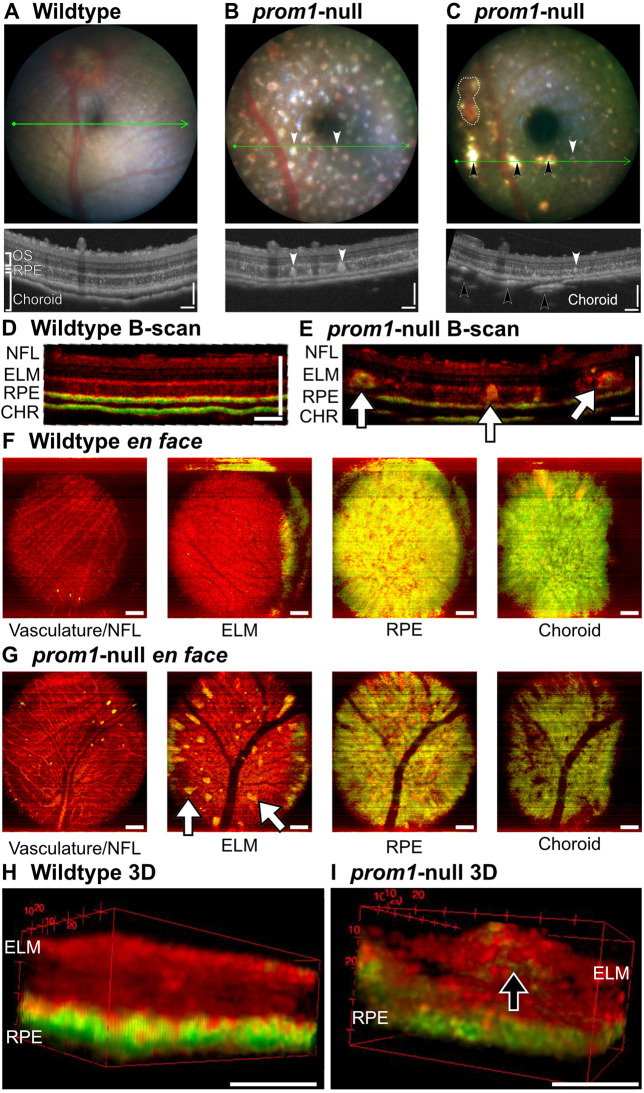
***prom1*-null mutations result in SDD-like deposits and RPE atrophy, assayed by color fundus photography and OCT.** (A–C) Representative images of *prom1*-null F1 frogs compared to those of wildtype animals in color fundus photography (top) and corresponding infrared optical coherence tomography (OCT) (bottom). F1 frogs had SDD-like deposits of cellular debris in the outer segment layer (B,C; white arrowheads). One phenotype had a large number of these deposits and no window defects or penetration of the OCT beam through the dense retinal pigment epithelium (RPE) layer and into the choroid (B), whereas the other had fewer SDD-like deposits and large window defects that revealed the underlying choroid (black arrowheads) and choroidal vasculature (dashed region, C). Green arrows on fundus images represent the corresponding OCT scan region, shown in the bottom images. Fundus field of view: 1.8 mm. Scale bars (OCT): 100 µm in *x* (horizontal bars) and *y* (vertical bars). (D–I) Comparison of B-scans (D,E), *en face* images (F,G) and 3D reconstructions (H,I) of a wildtype animal and a 3-year-old *prom1*-null adult frog with significant deposits using degree of polarization uniformity (DOPU) OCT. Deposits in *prom1*-null frogs had a significantly altered DOPU signal and were easily detectible as bright yellow or green spots in B-scan and *en face* orientations (E,G; white arrows). When projected in three dimensions, the DOPU signal was not uniform within the deposits. Instead, the green signal that indicates altered polarization caused by RPE pigment granules or other light-reflecting structures existed as a core within the deposit (I, black arrow). DOPU OCT detected loss of structural integrity of the external limiting membrane (ELM) as evidenced by the projection of the large deposits through this layer and loss of a strong band of red signal in the B-scan (E), *en face* images (G) and 3D reconstruction (I). RPE integrity breakdown is evidenced by breakdown of bright green bands in the B-scan (E) and patchiness in the yellow *en face* image (G). The large black vessels in the *en face* ELM, RPE and choroid images of the *prom1*-null animal (G) are not an anatomical defect but are instead caused by shadowing from the vitreal vessels blocking the DOPU OCT signal. Scale bars: 200 µm. NFL, nerve fiber layer; CHR, choroid. Number of animals: wildtype, *n*=6; *prom1*-null, *n*=9.

SDD-like deposits in *prom1*-null frogs were visible using color fundus photography and infrared optical coherence tomography (OCT) (Fig. 4A–C). An annotated image of retinal layers visible with OCT in *X. laevis* is shown in Fig. S2. In F1 animals, the deposits showed up in color fundus photography as blue-white or yellow-white lesions that formed a consistent mosaic pattern throughout the entire retina (Fig. 4B,C). This finding is consistent with the *X. laevis* retinal mosaic of nearly equally spaced rods (53%) and cones (47%) and lack of a macula or visual streak (Gábriel and Wilhelm, 2001). Some frogs also exhibited widow defects, which revealed the choroidal vasculature, in addition to hyper-reflective deposits (Fig. 4C). OCT confirmed that these deposits were located in the outer segment layer, between the photoreceptor inner segments and the apical side of the RPE (Fig. 4B,C). OCT segmentation and measurement indicated that *prom1*-null retinas were, on average, 13±2.9 µm thinner than those of wildtype frogs (*P*<0.05). The largest contributors to decreased retinal thickness were the outer segment layer (−3.5±1.6 µm, no significant difference) and the RPE (−7.9±1.0 µm, *P*<0.01) (Fig. S2).

SDD-like deposits were also visible using degree of polarization uniformity (DOPU) OCT as yellow-green lesions, indicating non-uniform polarization compared to that in surrounding retinal tissues (Fig. 4D–I). Wildtype animals had uniform bright yellow-green external limiting membrane (ELM), RPE and choroidal layers when examined with DOPU OCT (Fig. 4D,F), whereas *prom1*-null mutants with severe disease phenotypes and large numbers of deposits exhibited more patchiness in these layers (Fig. 4E,G). The largest of the SDD-like deposits had a ‘core’ of non-uniform polarization surrounded by uniform polarization (Fig. 4E,I) and they extended past the external limiting membrane, into the inner nuclear layer (Fig. 4E,G).

**Fig. 5.**
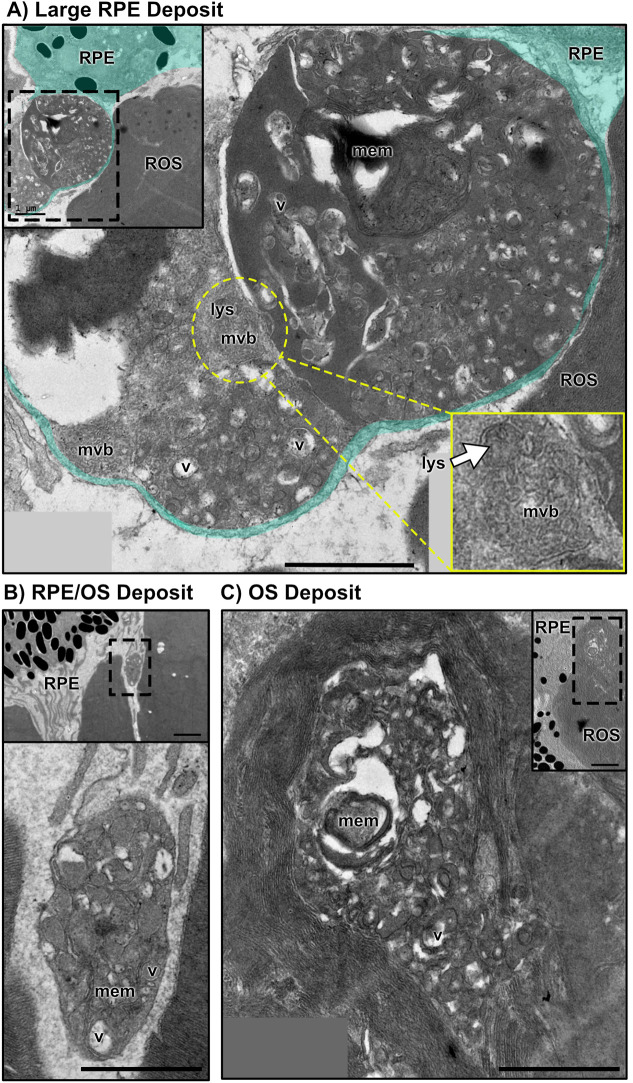
**Transmission electron micrographs of complex, lipofuscin-like deposits in the RPE of 8-month-old *prom1*-null mutants with a moderate disease phenotype.** These deposits were localized in (A,B) or near (C) the retinal pigment epithelium (RPE) and contained electron-dense material, membranes (mem), multivesicular bodies (mvb), lysosomes (lys) and numerous vacuoles (v). The yellow inset shows a higher magnification of a putative lysosome fusing to a disorganized multivesicular body in a large deposit located within the RPE. Scale bars: 0.5 µm; 1 µm (inset). ROS, rod outer segment.

In addition to patchiness and breakdown of the RPE as observed with light microscopy (Figs 2 and 3) and OCT (Fig. 4), transmission electron microscopy (TEM) of the RPE and outer segment tips of animals aged 8 months with moderate *prom1*-null disease revealed accumulations of complex, disorganized, electron-dense deposits that contained putative membranes, lysosomes, vacuoles and multivesicular bodies (Fig. 5). These bodies were located in or near the RPE and could represent lipofuscins or another type of indigestible waste by-product (Fig. 5A,B) as well as outer segment tip-shedding defects (Fig. 5C). To investigate the potential for defective disc shedding in *prom1*-null mutants further, we quantified the size and number of shed outer segment tips released shortly after dark adaptation. We observed that, as early as 2 weeks post fertilization, there were differences in outer segment shedding between *prom1*-null and wildtype animals. Shed membranes from *prom1*-null outer segment tips were smaller (4.2±0.5 µm^2^) and more numerous (27.7±5.1) than those shed by wildtype animals reared under the same conditions (9.1±2.1 µm^2^; 11.0±4.2; *P*<0.05) (Fig. S3).

**Fig. 6.**
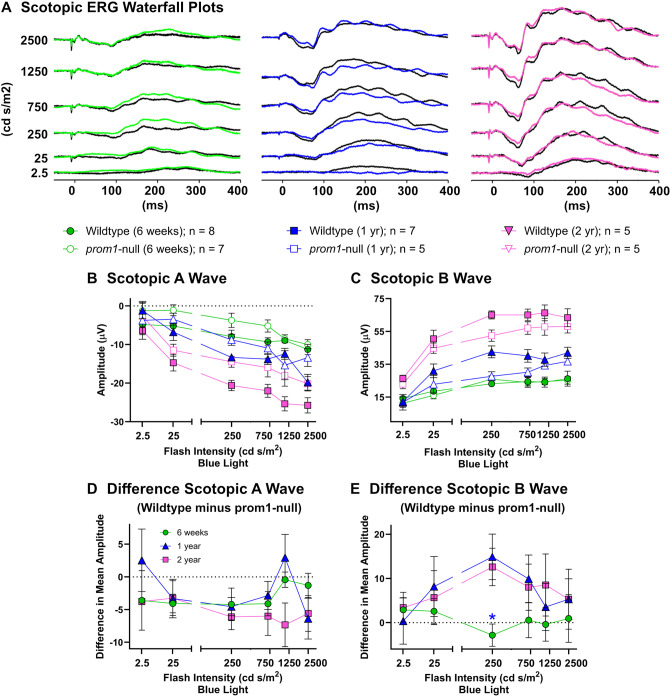
**Retinal function as measured by gross scotopic ERG response amplitude and the difference between wildtype and *prom1*-null animal response amplitude across three different ages.** (A) Waterfall plots of scotopic ERGs for 6-week-, 1-year- and 2-year-old frogs. Wildtype traces are black, whereas the mutant traces are colored green, blue and pink, respectively. (B,C) Scotopic A-wave (B) and B-wave (C) response amplitudes from wildtype and *prom1*-null animals. (D,E) The differences in A-wave (D) and B-wave (E) response amplitudes between wildtype and *prom1*-null animals. The numbers of animals and respective symbols are denoted under the waterfall plots in A. Difference data were calculated and graphed as the mean difference of wildtype minus *prom1-*null animals ±s.e.m. Data were analyzed using two-way ANOVA to segregate effects of age and light intensity. For difference data (D,E), statistical significance is denoted above the group that is different, with colors of asterisks indicating the statistical significance for the comparison group. * *P*<0.05. Data from 6-week-old animals have been published previously (Carr et al., 2021).

**Fig. 7.**
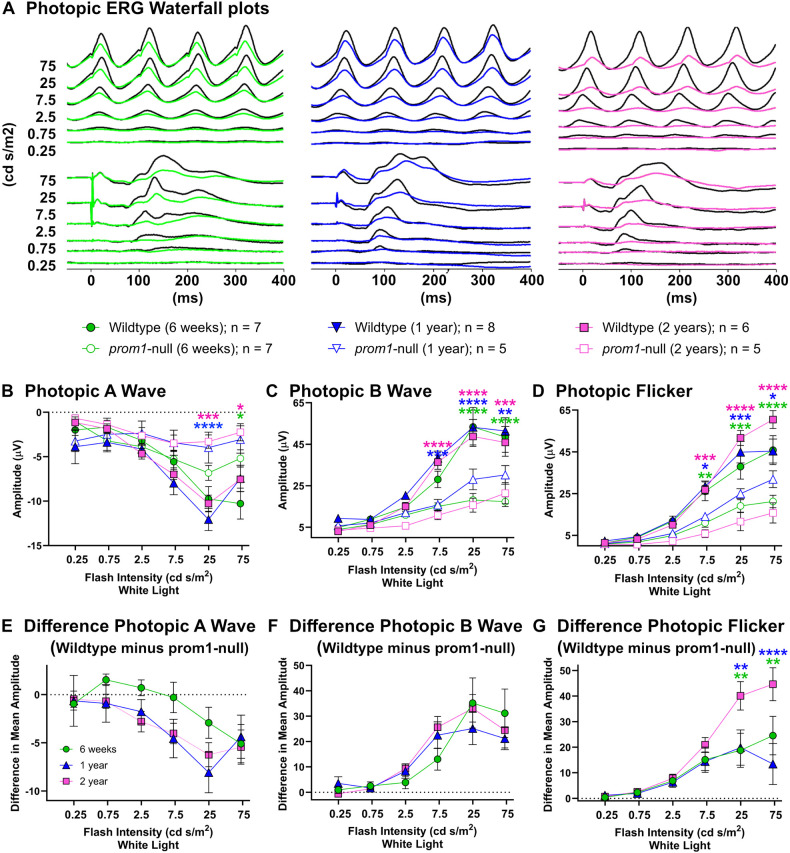
**Retinal function as measured by gross photopic ERG response and the difference between wildtype and *prom1*-null animal response amplitude across three different ages.** (A) Waterfall plots of photopic ERG and flicker responses for 6-week-, 1-year- and 2-year-old frogs. Wildtype traces are black, whereas the mutant traces are colored green, blue and pink, respectively. (B–D) Gross photopic A-wave (B), B-wave (C) and flicker (D) response amplitudes of wildtype and *prom1*-null animals. (E–G) The differences in photopic A-wave (E), B-wave (F) and flicker (G) response between wildtype and *prom1*-null animals. The numbers of animals and respective symbols are denoted under the waterfall plots in A. Difference data were calculated and graphed as the mean difference of wildtype minus *prom1*-null animals ±s.e.m. Data were analyzed by a two-way ANOVA with Tukey post hoc test. All statistical analysis data are listed in Table S1. For gross photopic amplitude response (B–D), statistical differences on the graphs denote differences between wildtype and *prom1*-null animals for the same age group; colors indicate the age. For difference data (E–G), statistical significance is denoted above the group that is different, with colors of asterisks indicating the statistical significance for the comparison group. *P<0.05, **P<0.01, ***P<0.001, ****P<0.0001. Data from 6-week-old animals have been published previously (Carr et al., 2021).

The scotopic electroretinogram (ERG) response of animals aged 6 weeks was slightly impaired, but not significantly, and there was no progressive impairment as animals aged to 1 and 2 years (Fig. 6A). There was no significant difference in A-wave amplitude between wildtype and *prom1*-null animals at any time point (Fig. 6B,D). The B-wave amplitude was slightly impaired in *prom1*-null animals at 1 and 2 years of age compared to 6 weeks; however, this difference was small and only statistically significant for 250 cd s/m^2^ between 6 weeks and 1 year of age (Fig. 6C,E; *P*<0.05). The photopic ERG was significantly impaired at 6 weeks, 1 year and 2 years of age, but it did not disappear completely, nor did this impairment continue to progress significantly between 6 weeks and 2 years of age for flash ERGs (Fig. 7A). The photopic A-wave was significantly impaired at all time points in *prom1*-null mutants compared to that in wildtype animals (Fig. 7B), but the difference between wildtype and *prom1*-animals remained the same up to 2 years (Fig. 7E). This was also true for the photopic flash B-wave; it was significantly impaired for *prom1*-null animals at all ages (Fig. 7C), but the impairment of the B-wave did not progress up to 2 years (Fig. 7F). The only significant progressive impairment observed between 6 weeks and 2 years of age was high-intensity photopic flicker (Fig. 7D,G). Two-year-old frogs had an increased impairment in flicker response at 25 (*P*<0.01) and 75 cd s/m^2^ (*P*<0.01) compared to 1-year-old and 6-week-old frogs, respectively. All statistical data for the two-way ANOVA are summarized in Table S1.

**Fig. 8.**
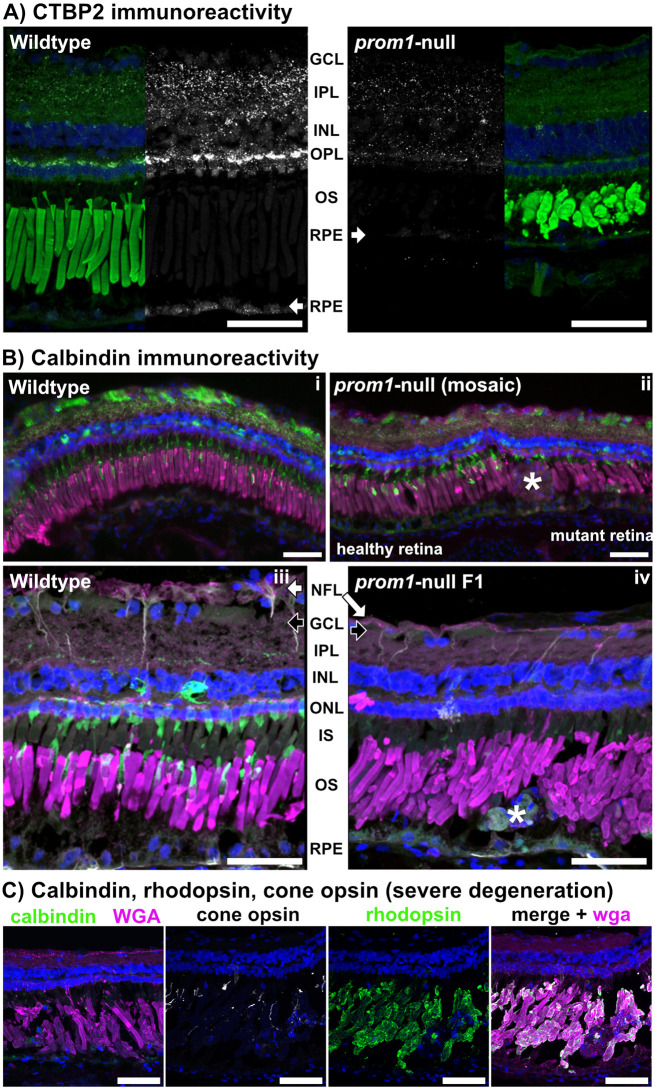
***prom1*-null frog retinas lose CTBP2 and calbindin labeling as they age.** Wildtype and *prom1*-null retinal immunoreactivity to CTBP2 (A), calbindin (B), and calbindin, rhodopsin and cone opsin (C). (A) There was significant loss of CTBP2 immunoreactivity in the central retinas and retinal pigment epithelium (RPE, white arrows) of *prom1*-null frogs aged 6 months. Staining for wheat germ agglutinin (WGA) is in green, CTBP2 in white and nuclei (Hoechst) in blue. (B) Compared to wildtype animals (i), in *prom1*-null animals aged ≥2 years (ii; mosaic F0 animal), there was significant loss of calbindin immunoreactivity (green) that started off as just loss in the cone outer segments, with calbindin labelling seen in healthy retina on the left, but loss of calbindin in mutant retina with an SDD-like deposit (ii, asterisk) on the right. This loss of calbindin expanded to immunoreactivity in a subset of bipolar cells concurrent with inner plexiform layer (IPL) and nerve fiber layer (NFL, white arrows) thinning (iv, F1 animal). The GCL is denoted by black arrows. There was also increased vimentin labeling (grey) in cellular debris deposits (iv, asterisk). Images in i,ii were acquired with a Leica DM600B epifluorescence microscope. Staining for calbindin (core inner segments and bipolar cells) is in green, WGA in magenta, vimentin in white and nuclei (Hoechst) in blue. (C) Severely degenerated retinas in *prom1*-null animals lost calbindin immunoreactivity completely and had significant thinning of the IPL. Some cone opsin immunoreactivity remained within the outer segment layer, but it was greatly reduced. Rhodopsin and WGA immunoreactivity were preserved. Scale bars: 50 µm. GCL, ganglion cell layer; INL, inner nuclear layer; IS, inner segments; ONL, outer nuclear layer; OPL, outer plexiform layer; OS, outer segments. Number of animals: wildtype, *n*=4; *prom1*-null, *n*=5.

To search for additional markers of retinal degeneration, we used C-terminal-binding protein 2 (CTBP2) to label photoreceptor and bipolar cell ribbon synapses. A major component of ribbon synapses is the protein RIBEYE, which comprises an A domain specific to ribbons and a B domain identical to CTBP2 (Schmitz et al., 2000). We also used calbindin to label cone inner segments and a subset of bipolar and amacrine cells (Morona et al., 2007). Decreased CTBP2 immunoreactivity was evident by 6 months post fertilization, before other obvious signs of retinal degeneration such as photoreceptor outer segment loss were seen, and was correlated with an increase in size and number of disorganized photoreceptor ribbon synapses in the cone pedicles of aging frogs as observed with TEM in 8-month-old frogs (Fig. 8A; Fig. S4). As animals aged greater than 2 years (3–6 years), worsening of outer segment disorganization and the presence of very large SDD-like deposits occurred concurrently with decreased calbindin immunoreactivity in the cone inner segments (Fig. 8B). Cone opsin immunopositivity was also decreased, although some scattered cone opsin-positive material remained adhered to the rod outer segments. Finally, severely degenerated and disorganized retinas exhibited loss of calbindin immunoreactivity in the cone inner segments and a subset of bipolar cells, accompanied by thinning of the inner plexiform layer (Fig. 8C). RPE thinning was not accompanied by changes in vitreal-choroidal vasculature as assayed using fluorescein angiography (Fig. S5).

## DISCUSSION

Here, we present evidence that *prom1*-null frogs develop SDD-like deposits of cellular debris in the outer segment layer and a cone-rod dystrophy phenotype. We also present data that support the hypothesis that retinal degeneration caused by *prom1*-null mutations is not due to direct effects on photoreceptor morphogenesis. It is instead a slow process associated with build-up of cellular debris in the outer segment layer and RPE atrophy, which is likely driven by RPE dysfunction and death prior to photoreceptor degeneration. These data suggest a novel mechanism of retinal degeneration for *prom1*-null mutations and demonstrate that *prom1*-null frogs might also be a suitable model organism for the study of dry AMD-related hallmarks such as deposit formation, metabolic waste build-up within the RPE, RPE atrophy and non-neovascular retinal atrophy.

Loss of Prom1 results in outer segment membrane overgrowth and loss of structural integrity (Fig. 1; Fig. S1) but not loss of membrane evaginations or inhibition of outer segment morphogenesis, as evidenced by long-term survival of the dysmorphic rods and cones reported here. Cones are more severely affected than rods (Carr et al., 2021) and *prom1*-null frogs eventually develop a cone-rod dystrophy phenotype evidenced by loss of calbindin immunoreactivity, thinning of the inner plexiform layer, and progressive decreases in photopic ERG flicker function, whereas rod outer segments express rhodopsin and the scotopic ERG response remains intact. We also detected changes in ribbon synapse immunoreactivity (Fig. 8A), size and morphology in *prom1*-null frogs with moderate to severe disease (Fig. S4). Similar findings are present in other animal models undergoing retinal remodeling in response to severe retinal degenerative disease such as retinitis pigmentosa, indicating a common endpoint of various classes of retinal degeneration (Jones and Marc, 2005). Similar to a previous report of *prom1*-null-associated retinal degeneration in mouse (Zacchigna et al., 2009), we saw no effect on vitreal blood vessel morphology or indicators of choroidal neovascularization. There were no morphological defects observed in the vitreal blood vessel structure by DOPU OCT (Fig. 4F) and there were no leaky vessels as assayed by fluorescein angiography (Fig. S5). In some older animals, choroidal vessels were faintly visible through the RPE when using angiography, but this was also observed in wildtype animals (Fig. S5). The frog retina is avascular, so vessels seen using fluorescein angiography are primarily the vitreal blood vessels (Miodoński and Bär, 1987). This phenotype is consistent with the autosomal recessive slow progressive retinal degeneration seen in human *PROM1*-null patients, who can take 15–50 years to develop a severe clinical phenotype (Gurudev et al., 2013). Extrapolating to the frog lifespan, which can be 15–20 years in captivity, aging of 2–4 years before significant retinal degeneration is analogous to the human clinical timeline and phenotype (Gurudev et al., 2013; Yang et al., 2008).

In addition to a cone-rod dystrophy phenotype, SDD-like deposits were present in aging *prom1*-null frogs and were visible in histology (Figs 1–3), color fundus photography (Fig. 4A–C) and as hyper-reflective foci using infrared and spectral domain OCT (Fig. 4D–I). Hyper-reflective foci are becoming an increasingly recognized biomarker for increased risk of severe retinal degenerative disease, but the definition of ‘hyper-reflective foci’ can refer to structures in the outer segment layer, the choroid or cystoid voids within the retina (Fragiotta et al., 2021). There are many hypotheses regarding the sources of material in the lesions, including macrophages, microglia, undigested lipid or protein material, retinal exudate or migrating RPE cells (Fragiotta et al., 2021). The deposits we observed in *prom1*-null frogs were localized to the outer segment layer, large (>30 µm) and not associated with retinal exudates, and did not exhibit OCT shadowing (Fig. 4B,C). This categorizes them as similar to hyper-reflective foci associated with severe outcomes of human retinal degenerative diseases such as retinitis pigmentosa (Kuroda et al., 2014), intermediate to severe AMD (Curcio et al., 2017) and Stargardt disease (Battaglia Parodi et al., 2019; Piri et al., 2015; Ritter et al., 2013). A limitation to the current study was that the OCT beam does not penetrate into the frog choroid as it does in the mammalian choroid (Gong et al., 2015), making it difficult to ascertain whether *prom1*-null frogs also have significant hyper-reflective foci in this layer. Notably, increasing size and number of hyper-reflective foci are considered a precursor of geographic atrophy (Christenbury et al., 2013; Leuschen et al., 2013; Schuman et al., 2009). Morphological features of progression to geographic atrophy include reduced retinal thickness, outer nuclear layer thinning and migration of hyper-reflective foci to the inner retina (Balaratnasingam et al., 2016; Ouyang et al., 2013).

Involvement of RPE dysfunction prior to photoreceptor degeneration in our *prom1*-null frogs is supported by several lines of evidence. Firstly, there was no progressive loss of ERG response from the rods and very little progressive loss of ERG response from the cones of *prom1*-null frogs between the ages of 6 weeks and 2 years (Figs 6 and 7). Outer segment membranes are synthesized continuously throughout life (Anderson et al., 1978; Young, 1967), and complete turnover of the outer segment membranes in *X. laevis* raised under normal lighting and temperature conditions should happen within approximately 28 days (Kinney and Fisher, 1978). Thus, if *prom1*-null mutations inhibited plasma membrane evaginations or some other process involved in outer segment membrane renewal, we would have expected to see significant loss of outer segments and loss of visual function on a significantly faster timescale (weeks to months). Instead, we observed significantly dysmorphic outer segments, indicating a role of the Prom1 protein in regulating membrane growth, but no loss of rod outer segments for ≥2 years.

The second line of evidence that retinal degeneration caused by *prom1*-null mutations is time- or age-dependent and involves the RPE is that visual impairment and retinal degeneration in *prom1*-null frogs was preceded by ever an increasing size and number of deposits of cellular debris in the subretinal space, which then extended further into the outer segment layer and eventually through the ELM into the inner retina (Figs 1–4). The deposits exhibited autofluorescence at significantly different wavelengths than those of the surrounding outer segment material, indicating a different source or significant protein and lipid degradation within the deposits. These deposits resembled SDDs in location and appearance by color fundus photography and OCT, and by their reaction to the histological stains Oil Red O and BODIPY (Fig. 2A–D). There are few animal models reported to faithfully recapitulate SDDs, and those that do require conditional double knockout of the cholesterol transporters ABCA1 and ABCG1 in macrophages (Ban et al., 2018).

In humans, SDDs most commonly present in the temporal superior quadrant of the macula, outside of the cone-dominated fovea (Curcio et al., 2013; Steinberg et al., 2015). Similarly, frog SDD-like deposits appear to be anatomically defined and are scattered across the retina in a regular mosaic pattern that matches the cone-rod photoreceptor mosaic (Fig. 4B,C). This distribution of SDD-like lesions associated with *prom1*-null mutations correlates with the idea that SDDs might be associated with a specific retinal cell type, namely rods, whereas soft drusen are associated with cones (Curcio et al., 2013). Although we were unable to detect any rhodopsin-positive immunoreactivity in the deposits, we were able to detect some cone opsin-positive labeling, which was restricted to honeycomb-like cell borders within the deposits, and not uniform throughout the deposit (Fig. 4A).

Taking into consideration the localization, OCT reflectivity and histology of the observed *prom1*-null frog SDD-like deposits, it appears likely that the cellular source of these deposits is the RPE. Evidence for this includes significant infiltration of RPE pigment granules into the deposits (Fig. 2A), disordered and strong RPE65-positive immunolabelling detected around ‘displaced nuclei’ (Fig. 2B) that could represent early stages in deposit formation, build-up of membranous and vacuolated material in and around the RPE as observed in TEM images (Fig. 5), and window defects that represent RPE and choroidal pigment loss in some *prom1*-null F1 animals (Fig. 4C). These window defects were best detected using DOPU OCT, which clearly showed the breakdown of highly polarizing signals in the pigmented RPE and choroid (Fig. 4G). Three-dimensional reconstruction of DOPU OCT signals in large deposits also showed a core of highly depolarizing signal surrounded by non-polarizing debris (Fig. 4I). This could represent a core of dead RPE material made up of depolarizing pigment granules, which continues to accumulate further debris as the animal ages. Finally, the retina or RPE of 2-year-old *prom1*-null animals were, on average, thinner than wildtype retinas as measured by OCT, and the RPE is the primary contributor to this effect (Fig. S2).

The RPE consists of terminally differentiated cells that are never replaced throughout the lifetime of the organism (Strauss, 2005). One of the primary functions of the RPE is to phagocytose shed outer segment membranes, creating a lifetime of significant oxidative stress as the lipid membranes are metabolized (Strauss, 2005). Oxidative stress can cause a process of cellular dysfunction and de-differentiation, termed the epithelial–mesenchymal transition (EMT) (Inumaru et al., 2009). EMT occurs when differentiated and polarized epithelial cells, such as the RPE, begin to lose their epithelial phenotype (polarized, adhesive, avascular) and begin to adopt a mesenchymal phenotype (non-polar, migratory, potentiating/self-renewing) (Inumaru et al., 2009). Some studies have reported that RPE cells can undergo EMT during severe retinal degenerative disease such as AMD (Datta et al., 2017). Characteristics associated with this change are increased migratory activity, invasiveness into the surrounding tissues, elevated resistance to apoptosis, and increased production of the extracellular matrix and cytoskeletal components such as vimentin, N-cadherin, β-catenin and fibronectin (Kalluri and Weinberg, 2009). We show that there is a similar pattern of relative increased vimentin in the large deposits in *prom1*-null frogs and in the RPE surrounding these deposits (Fig. 3C).

Another recent proposed function of PROM1 in the RPE is regulation of autophagy through the mTOR pathway in cell culture (Bhattacharya et al., 2017), where loss of PROM1 results in RPE EMT (Bhattacharya et al., 2023). Although we agree that loss of Prom1 results in EMT, as we see similar results in our *prom1*-null frogs, we did not observe Prom1 immunoreactivity in the differentiated frog RPE at any developmental time point (Fig. S6A,B). Moreover, RNA sequence expression data for human pan tissue plots (https://eyeintegration.nei.nih.gov/) show that, compared to the retina, where *PROM1* is known to be expressed, *PROM1* RNA expression is significantly lower in the RPE. It is also not present in the RPE cell lines ARPE-19, D407 or hTERT-RPE1, or primary RPE cultures (Fig. S6C). There can be high RNA expression in a tissue but no protein translation; however, there will not be high protein expression in a tissue that does not express RNA for the gene or protein of interest. Thus, we feel that it is unlikely that there is functional PROM1 protein controlling autophagy in fully differentiated RPE.

We propose that loss of Prom1 results in RPE EMT though a mechanism that involves increased phagocytic and oxidative stress caused by the dysmorphic *prom1*-null photoreceptor outer segments. As shown here and in our previous work (Carr et al., 2021), *prom1*-null outer segments lack structural integrity, especially the cones, and this loss of integrity could impact outer segment shedding, leading to an increased amount of material for the RPE to phagocytose and process (Fig. S3). Increased phagocytic load could cause oxidative stress and increased build-up of reactive oxygen and nitrogen species and indigestible material within the RPE and the SDD-like deposits. This is evidenced in our *prom1*-null frogs by positive E06 immunoreactivity within the deposits and surrounding RPE (Palinski et al., 1996), which is increased compared to that in wildtype retinas of the same age (Fig. 2E,F). Increased oxidative stress could then lead to EMT, causing even greater RPE dysfunction and creating a positive feedback loop of stress, damage and eventual RPE death. Effects corresponding to this were observed in the increased four-channel RPE autofluorescence (Fig. 1L), increased red autofluorescence (Fig. 2C,D), increased vimentin expression in the SDD-like deposits (Fig. 3C), and in the TEM images of the RPE that showed particles of undigested membrane and vacuole materials that were ≥1 µm in size (Fig. 5). This proposed mechanism is similar to the age-related build-up of lipofuscins and other materials in the RPE, which can also lead to decreased autophagic activity and RPE atrophy (Brunk and Terman, 2002). Accumulation of fluorescent metabolic byproducts of the visual transduction cycle and outer segment membrane phagocytosis is associated with photoreceptor degeneration and RPE death in Stargardt disease and atrophic (dry) AMD. This model also corresponds to the clinical manifestation of *PROM1*-associated retinal disease in humans, which is often diagnosed as cone-rod dystrophy, retinitis pigmentosa with cone involvement, or juvenile bull's eye macular degeneration (Permanyer et al., 2010; Pras et al., 2009; Zhang et al., 2007). Autosomal dominant *PROM1* disease (R373C) is often misdiagnosed as Stargardt disease before genetic testing occurs (Michaelides et al., 2010; Yang et al., 2008).

In summary, we show here that retinal degeneration in *prom1*-null frogs is slow and has a cone-rod dystrophy phenotype associated with SDD-like deposit formation and RPE atrophy. Based on these data, we hypothesize that the mechanism for recessive *prom1*-null associated blindness does not involve direct effects on photoreceptor morphogenesis, but instead likely involves dysfunction and death of the RPE that precedes photoreceptor degeneration. Finally, our *prom1*-null frogs showed analogous effects to some key hallmarks of dry AMD, including the presence of deposits, potential dysfunction and death of the RPE, accumulation of lipofuscins and autofluorescent material in the RPE, and non-neovascular retinal atrophy. Thus, further study and characterization of this model could also lead to significant findings relevant to AMD in addition to inherited blindness.

## MATERIALS AND METHODS

### Animal ethics statement, housing and anesthesia

Animal use protocols were approved by the University of British Columbia (A18-0257/A18-0259) and University of Alberta (AUP00004203) Animal Care Committees and carried out in accordance with the Canadian Council on Animal Care. Animals were housed at 18°C under a 12-h cyclic light schedule (900–1200 lux). For ERG and live-imaging experiments, adult frogs were anesthetized in 0.3% tricaine buffered in 0.1× MMR (10 mM NaCl, 0.2 mM KCl, 0.1 mM MgCl_2_·6H_2_O, 0.2 mM CaCl_2_, 0.55 mM HEPES) until non-responsive (8–15 min). Adult frogs were not anesthetized more than twice in one week and were allowed to recover for a minimum of 48 h between anesthetic exposures.

Frogs used in this study were a combination of male and female F0 and F1 animals that were created and characterized using the same methods as described previously (Carr et al., 2021). We did not see sex differences in disease phenotype, so data from all animals were pooled. CRISPR/Cas9 genetic modification was performed using a single-guide RNA (sgRNA) that targeted exon 1 of the *prom1.L* gene. Matings between animals that had confirmed indels by blood draw were performed to obtain F1 progeny. Data were semi-masked by analysis of immunofluorescence and ERG results before confirmation of genomic modification. Blood or skin samples were used for genomic DNA extraction to confirm and characterize CRISPR-mediated indels by Sanger sequencing subsequent to experimentation. Animals that did not have evidence of genomic editing by Sanger sequencing were excluded from final analysis for mutant groups. Previous studies indicated that *n*=6–10 is sufficient to detect differences between genetic groups (Carr et al., 2021). This was confirmed with a power calculation using G*Power and *t*-test parameters: two tails, effect size 0.75 (significant phenotype versus none), α error=0.05, β error=0.95. The total calculated sample size was 13 (six to seven animals per treatment group) (Faul et al., 2007).

### Immunofluorescence and Oil Red O staining

Whole eyes were fixed and prepared for immunohistochemistry as described previously (Carr et al., 2021). Tissues were mounted using medium prepared from Mowiol 4-88 (Mowiol mounting medium, 2006) (Millipore Sigma) and then visualized using a Zeiss LSM 800 microscope equipped with a 40× NA 1.2 water-immersion objective, a Zeiss LSM 880 with Airyscan equipped with a 63×1.4 NA oil-immersion objective, or a Leica DM600B epifluorescence microscope with a Leica K3M 6.3 MP CMOS camera. Micrographs represent maximum-intensity projections of whole retinal sections (*z*=0.3–0.5 µm/step) or epifluorescence micrographs. Antibody, histology stain and lectin sources and concentrations used in this study are listed in Table S2.

Oil Red O (Millipore Sigma) was freshly prepared each time. 0.5% (w/v) Oil Red O was dissolved in 100% isopropanol, filtered with a syringe filter (0.22 µm) and then mixed as three parts Oil Red O to two parts dH_2_O to prepare a 0.3% (w/v) staining solution diluted in 60% isopropanol. Tissue sections were washed three times for 8 min in PBS to remove optimal cutting temperature (OCT) compound (Thermo Fisher Scientific), incubated in 60% isopropanol for 5 min, and then stained with 0.3% Oil Red O for 15 min. Stained tissues were rinsed two to five times (until clear) with 60% isopropanol using a transfer pipette and then washed three times for 8 min in PBS with gentle shaking. Tissues were then coverslipped using Mowiol mounting medium (Mowiol mounting medium, 2006) and imaged in brightfield using the Zeiss LSM 800 confocal microscope and objectives as described above. A Zeiss Axiocam 503 color camera was used to collect brightfield images.

### TEM

Detailed methods for tissue preparation for TEM followed previously published protocols (Carr et al., 2021; Tam et al., 2015). Whole eyes were fixed in 4% paraformaldehyde and 1% glutaraldehyde diluted from 20% and 8% stock solutions, respectively (Electron Microscopy Sciences, Hatfield, PA, USA). Eyes were fixed at 4°C for ≥24 h and then infiltrated with 2.3 M sucrose, embedded in OCT compound, and cryosectioned at 20 µm using a Leica CM1860 cryostat with single sections per slide. Optimally oriented sections were washed with 0.1 M sodium cacodylate and then stained with 1% osmium tetroxide for 30 min. After staining, sections were dehydrated in increasing concentrations of anhydrous ethanol (30, 50, 70, 90 and 95%, each for 5 min; 100%, three washes for 5 min) and then infiltrated with increasing concentrations of Eponate 12 resin (Ted Pella, Redding, CA, USA) diluted with anhydrous acetone (35, 50, 70%, each for 40 min; 100%, twice for 40 min). Beem capsules with the ends trimmed off were placed over the fully infiltrated sections and then the capsule was filled with resin and allowed to polymerize overnight. Ultrathin silver-grey sections (∼50–70 nm) were cut with a diamond knife using a Reichert UltraCut E ultra-microtome and collected on Formvar-coated nickel slot grids (Electron Microscopy Sciences). Sections were stained with saturated aqueous uranyl acetate and Venable and Coggeshall's lead citrate (Venable and Coggeshall, 1965). Imaging was performed with a JEOL 2100 electron microscope at 200 kV. High-resolution TEM images (Fig. 5) were created by capturing overlapping high-magnification images and then stitching them together using the Adobe Photoshop automatic photomerge function. Images were then verified manually to ensure that the stitching was accurate.

### Optical coherence tomography, fundus photography and fluorescein angiography

Infrared OCT and color fundus images were taken using a Micron IV system with the mouse-specific objective lens (Phoenix Technology Group, Pleasanton, CA, USA). Brightfield color fundus images had a field of view of 1.8 mm. The infrared OCT spectrometer covered 750–930 nm and the full line-scan size was 1.6 mm in *x* and *y* coordinates. Micron IV OCT images were created from 15 averaged segments. For fluorescein angiography, 25% fluorescein sodium (Millipore Sigma) diluted in sterile dH_2_O was injected into the dorsal lymph sac (2 µl/g of frog). The initial concentration was based on previous studies in mouse (Chang, 2013) and then optimized empirically for this study. The resulting retinal blood vessel fluorescence was imaged using the Micron IV color fundus camera at its peak intensity, 20–30 s after injection.

Spectral-domain OCT was performed with a custom-built system tailored for small-animal retinal imaging (Wahl et al., 2019). The system utilized a superluminescent diode laser source with a center wavelength of 840 nm and a full-width-half-maximum bandwidth of 100 nm yielding axial resolution of approximately 3.1 µm. The detection of the OCT signal was performed by two separate custom-built spectrometers with a maximum A-scan rate of 250 kHz, which were numerically calibrated to acquire identical back-scattering signals from the subject with an opposite polarity (Miao et al., 2022). The optical signals acquired from the spectrometers were processed to extract melanin granule-specific contrast from the subject by estimating a noise-suppressed DOPU, for which the processing algorithm adhered to a protocol developed previously (Hsu et al., 2020; Makita et al., 2014). Each processed multi-contrast DOPU OCT image presented in this study was constructed with 450 A-scans per B-scan and 500 B-scans per volume.

### ERG

ERGs were recorded using electrodes connected to a model 1800 AC amplifier and head stage (A-M Systems, Sequim, WA, USA) and an Espion Ganzfeld stimulator (ColorDome) and recording unit (Diagnosys, Lowell, MA, USA) as described previously (Vent-Schmidt et al., 2017) with minor modifications as follows. The ground, a flat gold EEG electrode, was glued into a 100 mm Petri dish instead of a 60 mm Petri dish. The chin of the adult frogs was placed on the ground electrode. To accommodate the rest of the body, a third of the sides of the 100 mm Petri dish were cut off, smoothed with sandpaper, and then lined with soft weather stripping to prevent scratching the belly of the frogs and to keep the frogs from sliding out of the dish during ERG recordings. Corneal recordings were made from a chlorinated silver wire electrode set in a pulled glass micropipette and mounted into an electrode pin holder (A-M Systems). The entire electrode assembly was mounted in a manual micromanipulator (US-3F, Narishige International, Amityville, NY, USA). Adult frogs were anesthetized in 0.1% tricaine in 0.1× MMR until they were just unresponsive, as deep anesthesia can negatively impact the strength of the ERG response (Nair et al., 2011). Scotopic ERGs were recorded in animals that had been dark-adapted overnight and prepared under dim red light; recorded stimuli were the average of five trials. Photopic ERGs were recorded in animals that had been exposed to a normal light cycle and prepared under regular lab lighting (∼350 lux); recorded stimuli were the average of ten trials. For scotopic and photopic ERG, the A-wave amplitude was measured as the minimum value (trough) between 50–150 ms post-flash stimulus. The B-wave amplitude was measured as the value from the a-wave trough to the b-wave peak, which was defined as the maximum value measured between 100–250 ms post-flash stimulus. Flicker amplitude was measured as the average of the values from trough to peak for five cycles.

ERG waveforms were visualized using Excel (Microsoft) and analyzed by measuring and plotting the A-wave and B-wave peak amplitudes, and then fitting the resulting curves using non-linear regression analysis. Genotype effects, light intensity effects and interaction effects were analyzed by two-way ANOVA with Sidak's post hoc test (Table S1).

### Image analysis for Prom1 immunoreactivity and shed outer segment tips

Prom1 immunolabeling signal intensity was measured in FIJI (Schindelin et al., 2012) using a macro created by Dr Elizabeth Kugler (Zeeks – Art for Geeks Ltd; EKugler_11082021.ijm). Stitched micrographs were acquired using the same image settings and then raw data files were imported into FIJI. Thresholding was applied to the WGA channel, which facilitated automatic drawing of outer-segment regions of interest (ROIs). Thresholded ROIs were used to measure summed pixel intensity (raw integrated density) in both the WGA and Prom1 channels, and the Prom1 signal was then normalized to the WGA signal for each ROI using the formula: (Prom1 signal/WGA signal)×100. All normalized ROI calculations were averaged for each age group and plotted in GraphPad Prism (V10.2.2; San Diego, CA, USA). Because data were not normally distributed, a Mann–Whitney U test was used to compare overall Prom1/WGA normalized signal intensity.

To quantify outer segment disc shedding, tadpoles were raised completely in the dark for 2 weeks. On the morning of day 15 (around 10:00), tadpoles were exposed to 10–15 min of laboratory light (∼500 lux), after which the animals were euthanized, the eyes immediately fixed, and then the tissues were processed for immunohistochemistry as described above. To count the size and number of shed tips, ROIs were manually drawn around visible shed outer segment tips in fluorescence micrographs of the central retina representing a field of view of 160 µm×160 µm. The ROI number and area was calculated using the measure function in FIJI (Schindelin et al., 2012).

### Software

Statistical analysis and graphing were performed using GraphPad Prism. Micrographs were analyzed and figures constructed using Affinity Photo (Serif, West Bridgford, UK) and FIJI.

## Supplementary Material



10.1242/joces.262298_sup1 Supplementary information
