# Supplementary material for: prominin-1-null Xenopus laevis develop subretinal drusenoid-like deposits, cone-rod dystrophy and RPE atrophy
Source: J Cell Sci. 2024 Nov 12;137(21):jcs262298. doi: 10.1242/jcs.262298 (PMC11586525; doi:10.1242/jcs.262298)
Supplement: Supplementary information [file joces-137-262298-s1.pdf]

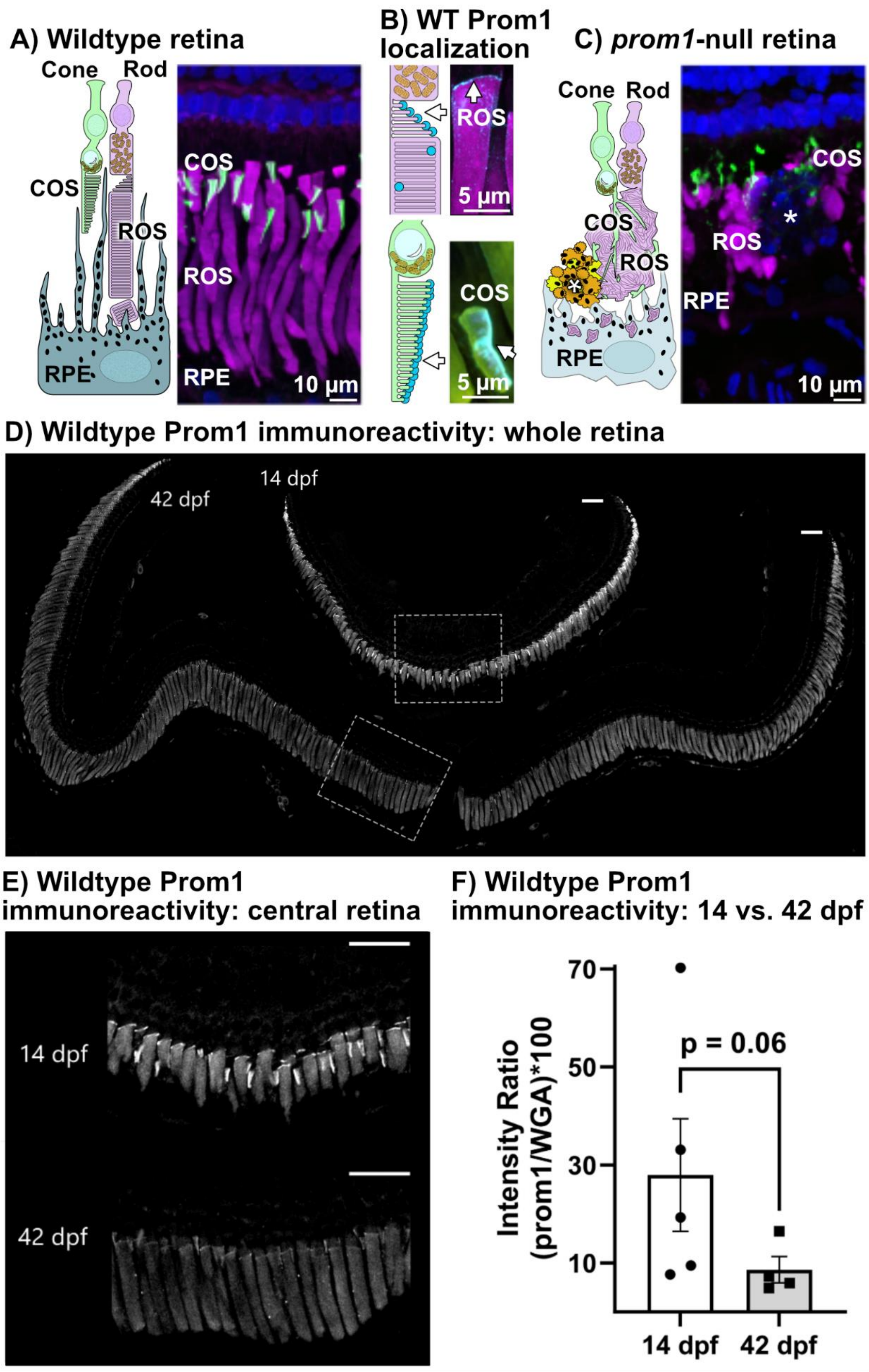

Journal of Cell Science • Supplementary information

Fig. S1. Schematic of photoreceptor/RPE morphology and Prom1 protein localization and expression in wildtype and *prom1*-null frogs. A) Wildtype outer segment morphology is highly organized in both rods and cones. Rods (magenta) comprise discrete discs of membrane whereas cones (green) comprise layered lamellae. B) In wildtype frogs, Prom1 protein (cyan) is localized to the base of the rod outer segment (magenta) and one side of the cone outer segment (green). C) Outer segment morphology in *prom1*-null frogs is significantly dysmorphic; ROS are shortened and bulbous, COS are fragmented. There are also deposits of heterogeneous cellular material between the OS and RPE (asterisk). D-F) Prom1 protein immunoreactivity in wildtype animals aged 14- and 42-days post fertilization. Relative Prom1 immunoreactivity decreases in the central retina as animals age and outer segments reach adult size. Scale bar = 50 µm. Bar graph demonstrates mean ± SEM. *Number of animals:* 14 dpf, n = 5; 42 dpf, n = 4. *Micrograph labels:* Magenta, wheat germ agglutinin; Green, cone opsin; blue, Hoechst 33342 nuclear stain; cyan/grey, Prom1. *Abbreviations:* COS, cone outer segment; dfp, days post fertilization; ROS, rod outer segment; RPE, retinal pigment epithelium.

Journal of Cell Science • Supplementary information

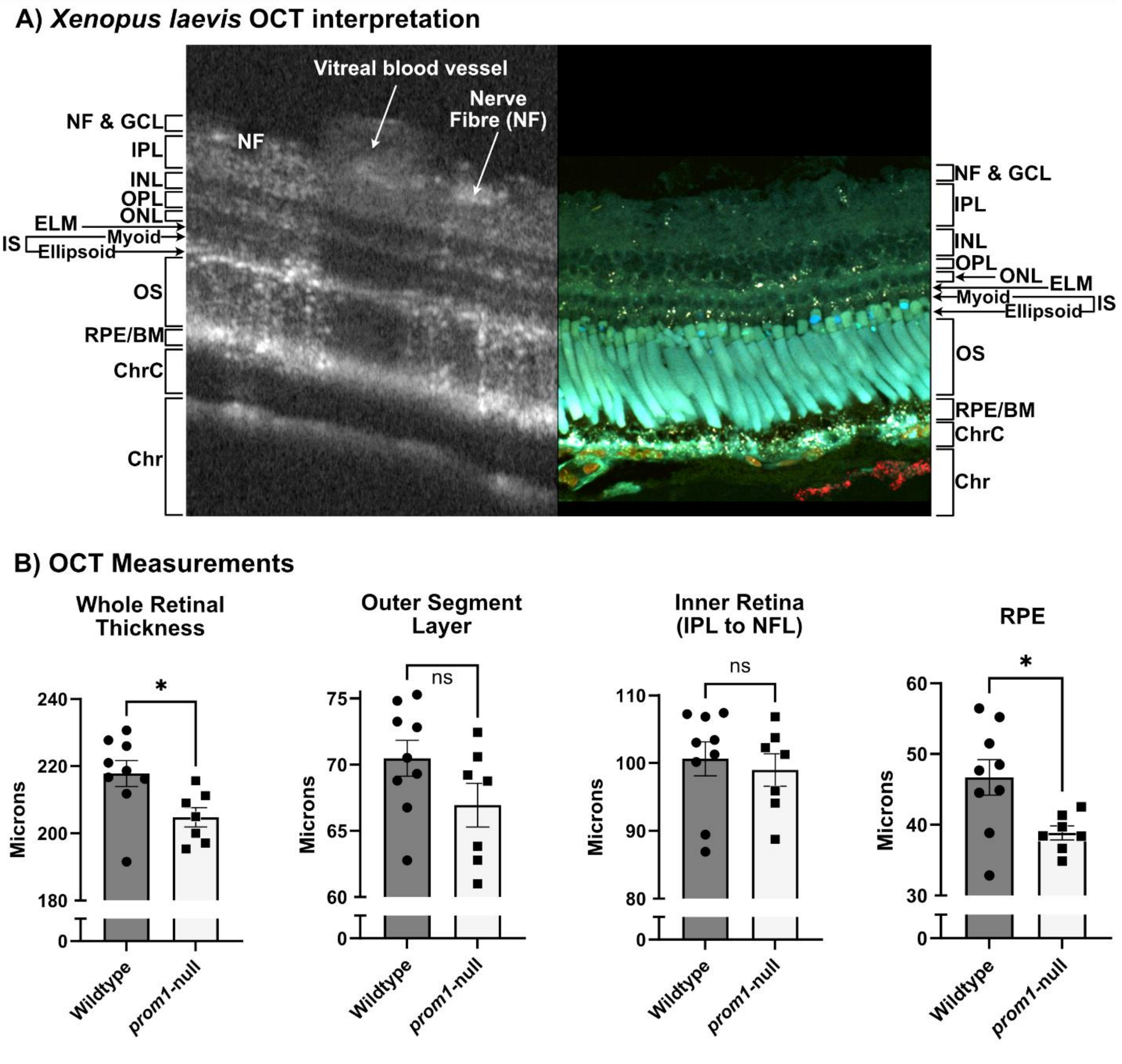

Journal of Cell Science • Supplementary information

Fig. S2. OCT interpretation and measurement of retinal layer thicknesses in wildtype and *prom1*-null frogs. A) OCT interpretation of wildtype *Xenopus laevis* retina. B) Retinal layer thickness as measured by OCT in 2-year-old wildtype and F0 *prom1*-null animals. The RPE represented the greatest contributor to overall thinning of the retinal in *prom1*-null frogs. Graphs represent the means ± SEM. *Number of animals:* Wildtype, n = 9; *prom1*-null, n = 7.

Journal of Cell Science • Supplementary information

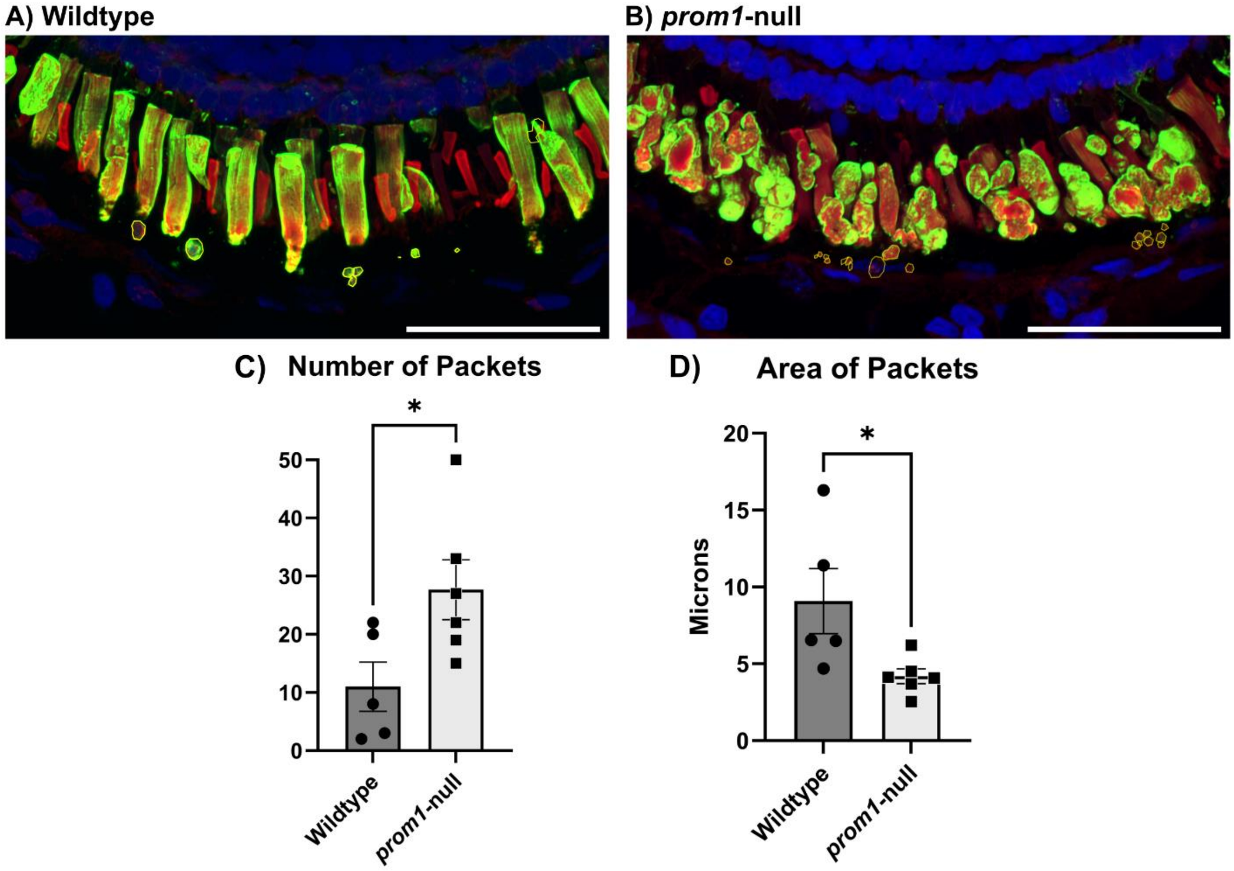

Fig. S3. **Comparison of the size and number of shed outer segment packets in wildtype versus *prom1*-null mutant tadpoles aged 14 dpf.** Scale bars = 50 µm. Data are represented as mean ± SEM. *Number of animals:* wildtype, n = 5; *prom1*-null n = 6. *Statistics:* Student's t-test, unpaired, two-tailed; * $p < 0.05$.

Journal of Cell Science • Supplementary information

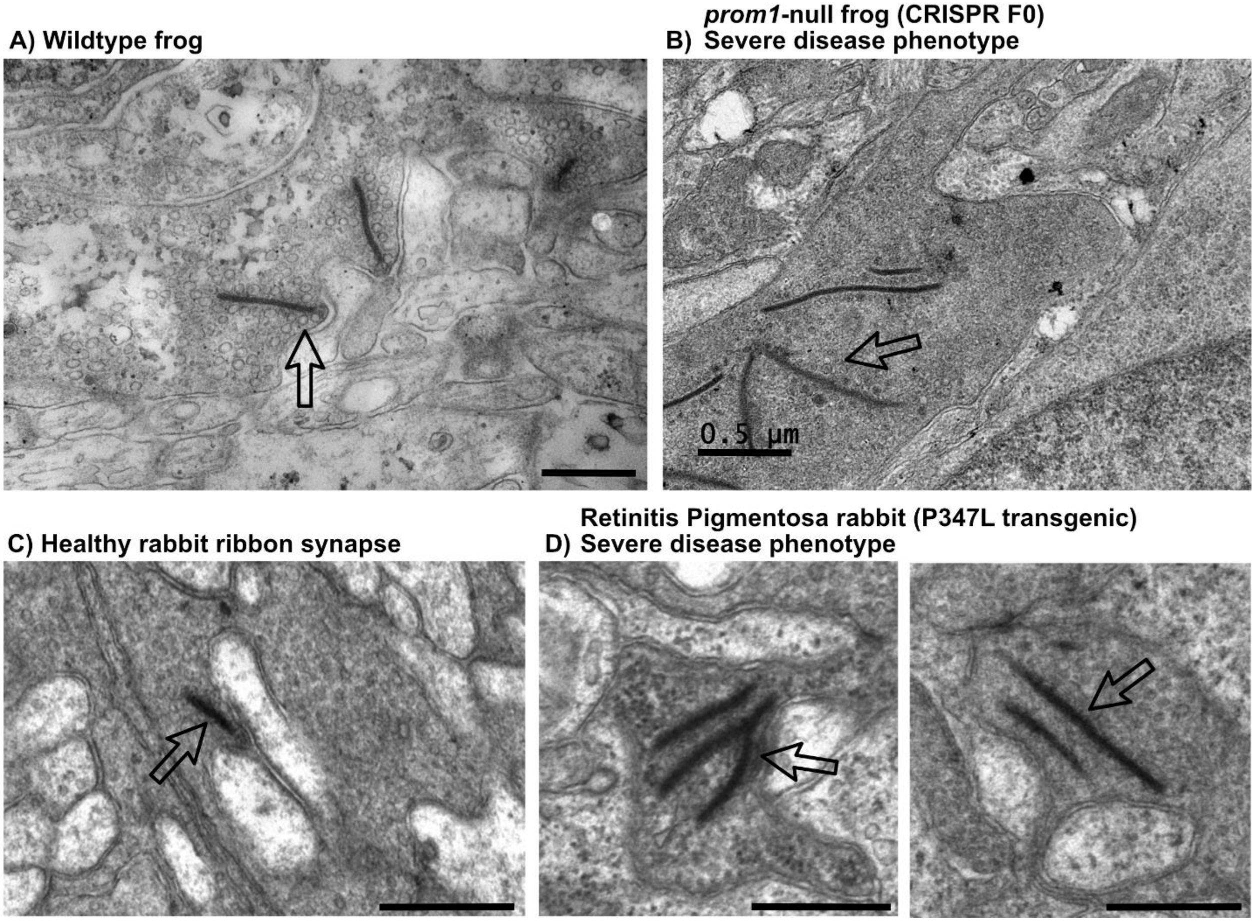

Fig. S4. A comparison of morphology changes in photoreceptor synapses between a wildtype frog, a *prom1*-null frog, and a rabbit model of retinitis pigmentosa (P347L). Rabbit ultrastructural volumes were accessed using the Retinal Pathoconnectome 1 (RPC1) dataset from the Viking Database (Anderson et al., 2011; Pfeiffer et al., 2020), funded by National Institutes of Health grant EY028927 (Bryan W. Jones, University of Utah School of Medicine, Salt Lake City, Utah, USA, 84132). Remodelling features are very similar between the two animals and the two models of retinal degeneration, indicating similarities in the downstream processes of severe retinal degeneration. Scale bar = 0.5 µm.

Journal of Cell Science • Supplementary information

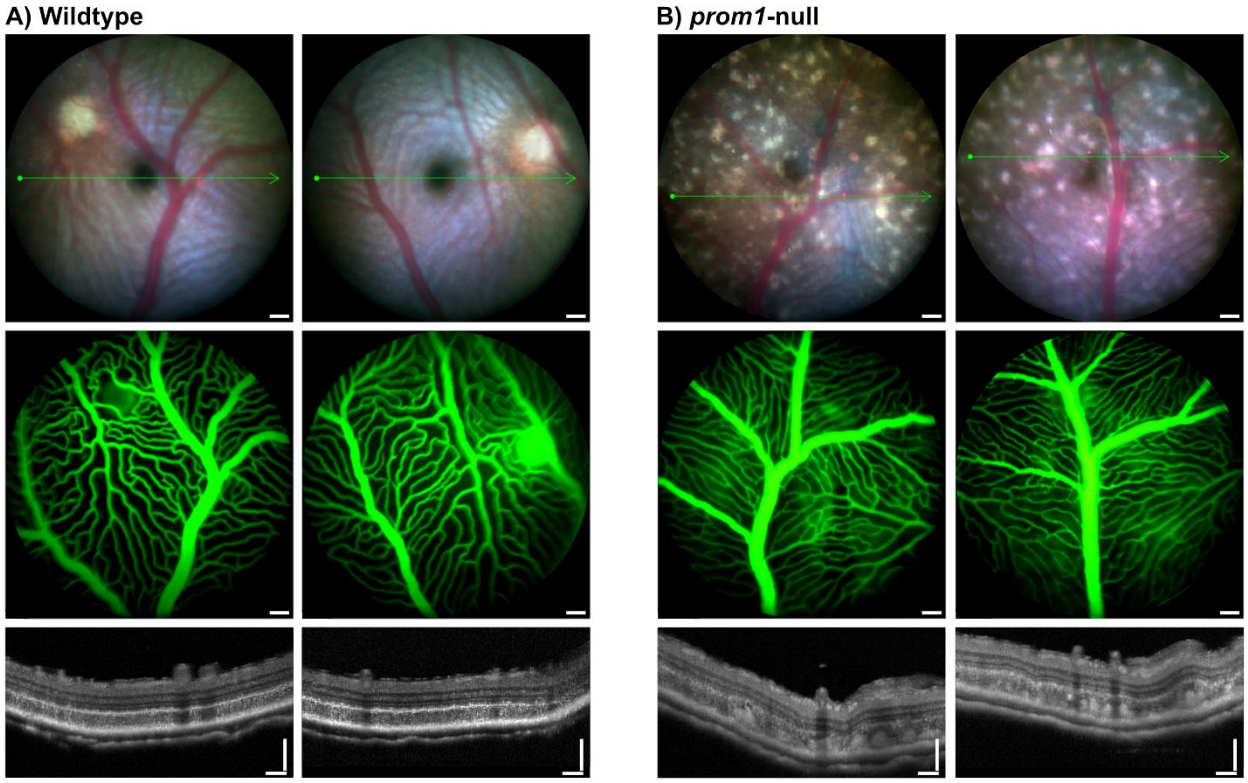

Fig. S5. **Fluorescein angiography in 3-year-old wildtype and *prom1*-null mutant frogs.** There was little observed difference in the vitreal blood vessel structure and there were no leaky vessels or significant bleed through to the choroidal vessels near the large lesions. Scale bars = 100 µm. *Number of animals:* Wildtype, n = 9, *prom1*-null, n = 8.

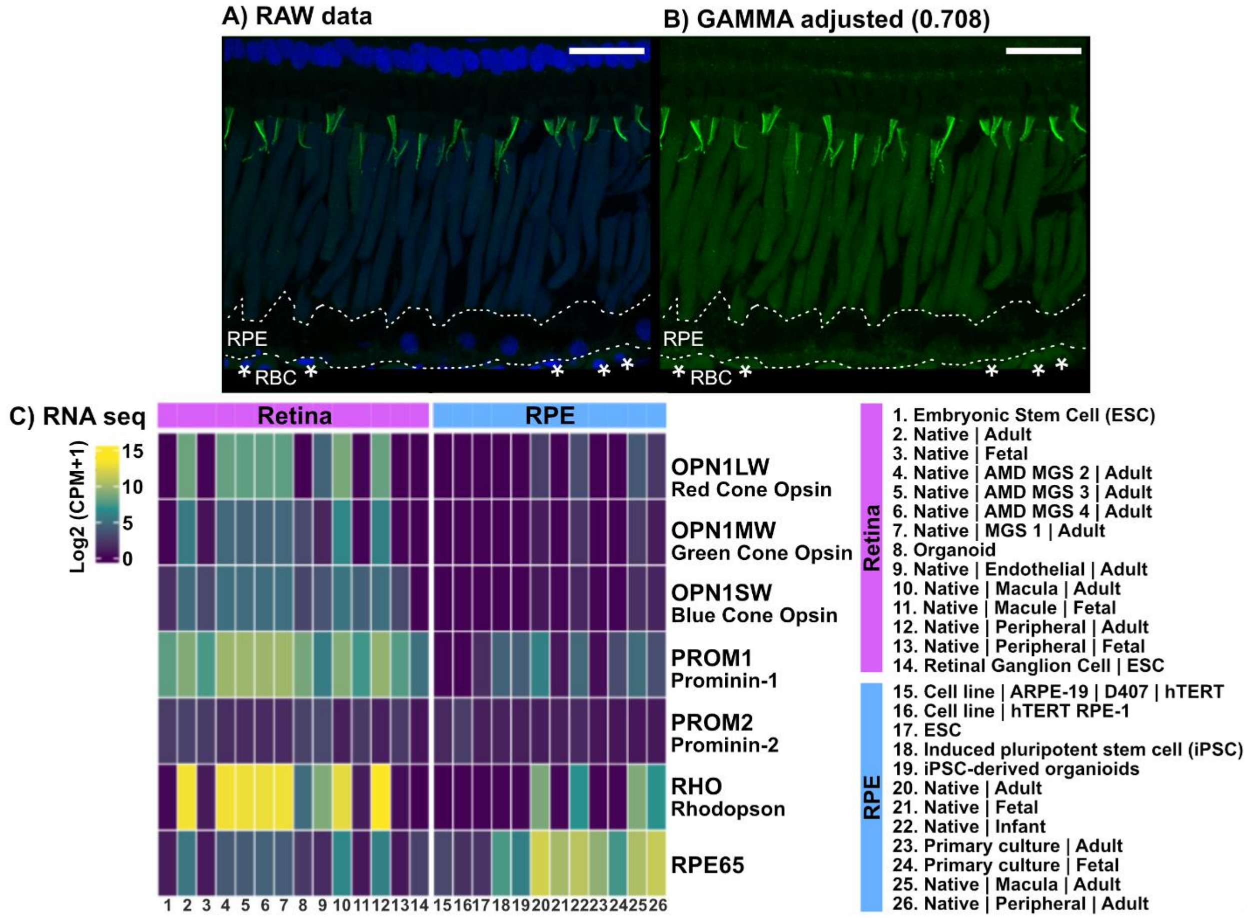

Fig. S6. **Lack of Prom1 protein immunoreactivity in fully differentiated frog RPE and human pan retinal/RPE RNA seq data from the NEI eyeIntegration Database**[1–15] v2.12 (https://eyeintegration.nei.nih.gov/). A) Representative micrograph of Prom1 immunoreactivity in an adult frog retina and RPE. There is no detectable Prom1 immunoreactivity in the RPE (white dotted lines). Red blood cell nuclei are denoted with an asterisk. B) GAMMA adjusted micrograph to increase the overall intensity of the Prom1 protein signal against background to demonstrate lack of immunoreactivity in the RPE. *Number of animals*: n = 63. C) A heat map of RNA seq expression data for cone opsins, *PROM1*, *PROM2*, *RHO*, and *RPE65* in the retina/RPE. Samples are denoted by number at the bottom of the heat map and identified by the legend on the right. *Abbreviations:* RPE, retinal pigment epithelium; RBC, red blood cells.

Table S1. Statistics for photopic flash and flicker ERG. *Asterisks:* * $p < 0.05$, ** $p < 0.01$, *** $p < 0.001$, **** $p < 0.0001$.

**Photopic A Wave**	**Wildtype vs. *prom1*-null**			
Light Intensity (cd s/m2)	6 weeks	1 year	2 years	**Two-Way ANOVA**
0.25	ns	ns	ns	Interaction Effect: p = 0.0034
0.75	ns	ns	ns	Simple Main Light Intensity Effect: p < 0.0001
2.5	ns	ns	ns	Simple Main Genotype Effect: p < 0.0001
7.5	ns	ns	ns	
25	ns	****	***	
75	*	ns	*	

**Photopic B Wave**	**Wildtype vs. *prom1*-null**			
Light Intensity (cd s/m2)	6 weeks	1 year	2 years	**Two-Way ANOVA**
0.25	ns	ns	ns	Interaction Effect: p < 0.0001
0.75	ns	ns	ns	Simple Main Light Intensity Effect: p < 0.0001
2.5	ns	ns	ns	Simple Main Genotype Effect: p < 0.0001
7.5	ns	***	****	
25	****	****	****	
75	****	**	***	

**Photopic Flicker**	**Wildtype vs. *prom1*-null**			
Light Intensity (cd s/m2)	6 weeks	1 year	2 years	**Two-Way ANOVA**
0.25	ns	ns	ns	Interaction Effect: p < 0.0001
0.75	ns	ns	ns	Simple Main Light Intensity Effect: p < 0.0001
2.5	ns	ns	ns	Simple Main Genotype Effect: p < 0.0001
7.5	**	*	***	
25	***	***	****	
75	****	*	***	

Journal of Cell Science • Supplementary information

**Diff. Photopic Flicker**	**Wildtype minus *prom1*-null**			
Light Intensity (cd s/m_2)	6 weeks vs. 1 yr	6 wks vs. 2 yrs	1 yr vs. 2 yrs	**Two-Way ANOVA**
0.25	ns	ns	ns	Interaction Effect: p = 0.0165
0.75	ns	ns	ns	Simple Main Light Intensity Effect: p < 0.0001
2.5	ns	ns	ns	Simple Main Genotype Effect: p = 0.0006
7.5	ns	ns	ns	
25	ns	**	**	
75	ns	**	****	

Journal of Cell Science • Supplementary information

Table S2. Antigens, species/type, source, and working dilutions of antibodies used in this study.

**Primary Antibodies**			
**Antigen**	**Species/Type**	**Source**	**Concentration**
X. laevis prominin-1 (N-terminus)	rabbit polyclonal	D.S. Papermaster[16,17]	1:750
X. laevis cone opsin (red-sensitive)	rabbit polyclonal	O.L. Moritz[17]	1:500
X. laevis rhodopsin (B6-30N)	mouse monoclonal	W. Clay Smith & Paul Hargrave[18]	0.6 µg/mL
X. laevis vimentin	mouse monoclonal	DSHB (14h7)	7.6 µg/mL
CTBP2	rabbit polyclonal	Abcam (ab151766)	1.5 µg/mL
Calbindin D-28K	rabbit polyclonal	Sigma-Aldrich (PC253L)	1:500
Anti-Oxidized phospholipid [E06][19]	mouse monoclonal	Absolute Antibody Ltd (Ab02746-21.0)	2.5 µg/mL
**Secondary Antibodies**			
AF488	anti-mouse<br>anti-rabbit	Jackson ImmunoResearch	2 µg/mL
Cy3	anti-mouse<br>anti-rabbit	Jackson ImmunoResearch	2 µg/mL
**Lectins**			
**Target**	**Fluorophore**	**Source**	**Concentration**
Wheat germ agglutinin (WGA)	AF488, AF594, AF647	Life Technologies	2-50 µg/mL
**Stains**			
**Stain**	**Target**	**Source**	**Concentration**
Hoechst 33342	nuclear stain	Sigma Aldrich	0.1 mg/mL
Oil Red O	neutral lipids	Sigma Aldrich	0.3% (w/v)
BODIPY 493/503	neutral lipids	Cayman Chemical	10 µg/mL

Journal of Cell Science • Supplementary information

SUPPLEMENTARY REFERENCES

1. Au, K. F., Jiang, H., Lin, L., Xing, Y. & Wong, W. H. Detection of splice junctions from paired-end RNA-seq data by SpliceMap. *Nucleic Acids Research* **38**, 4570–4578 (2010).
2. Bryan, J. M. *et al.* Identifying core biological processes distinguishing human eye tissues with precise systems-level gene expression analyses and weighted correlation networks. *Human Molecular Genetics* **27**, 3325–3339 (2018).
3. Darrow, E. M. *et al.* Deletion of *DXZ4* on the human inactive X chromosome alters higher-order genome architecture. *Proc. Natl. Acad. Sci. U.S.A.* **113**, (2016).
4. Farkas, M. H. *et al.* Transcriptome analyses of the human retina identify unprecedented transcript diversity and 3.5 Mb of novel transcribed sequence via significant alternative splicing and novel genes. *BMC Genomics* **14**, 486 (2013).
5. Gill, K. P. *et al.* Enriched retinal ganglion cells derived from human embryonic stem cells. *Sci Rep* **6**, 30552 (2016).
6. Hu, G. *et al.* Identification of miRNA Signatures during the Differentiation of hESCs into Retinal Pigment Epithelial Cells. *PLoS ONE* **7**, e37224 (2012).
7. Kaewkhaw, R. *et al.* Transcriptome Dynamics of Developing Photoreceptors in Three-Dimensional Retina Cultures Recapitulates Temporal Sequence of Human Cone and Rod Differentiation Revealing Cell Surface Markers and Gene Networks. *Stem Cells* **33**, 3504–3518 (2015).
8. Li, M. *et al.* Comprehensive analysis of gene expression in human retina and supporting tissues. *Human Molecular Genetics* **23**, 4001–4014 (2014).
9. Mustafi, D. *et al.* Transcriptome analysis reveals rod/cone photoreceptor specific signatures across mammalian retinas. *Hum. Mol. Genet.* ddw268 (2016) doi:10.1093/hmg/ddw268.
10. Peng, S. *et al.* Engineering a Blood-Retinal Barrier With Human Embryonic Stem Cell-Derived Retinal Pigment Epithelium: Transcriptome and Functional Analysis. *Stem Cells Translational Medicine* **2**, 534–544 (2013).
11. Pinelli, M. *et al.* An atlas of gene expression and gene co-regulation in the human retina. *Nucleic Acids Res* **44**, 5773–5784 (2016).
12. Santaguida, S., Vasile, E., White, E. & Amon, A. Aneuploidy-induced cellular stresses limit autophagic degradation. *Genes Dev.* **29**, 2010–2021 (2015).
13. Tresini, M. *et al.* The core spliceosome as target and effector of non-canonical ATM signalling. *Nature* **523**, 53–58 (2015).

Journal of Cell Science • Supplementary information

14. UK10K Consortium *et al.* An siRNA-based functional genomics screen for the identification of regulators of ciliogenesis and ciliopathy genes. *Nat Cell Biol* **17**, 1074–1087 (2015).
15. Whitmore, S. S. *et al.* Transcriptomic analysis across nasal, temporal, and macular regions of human neural retina and RPE/choroid by RNA-Seq. *Experimental Eye Research* **129**, 93–106 (2014).
16. Han, Z., Anderson, D. W. & Papermaster, D. S. Prominin-1 localizes to the open rims of outer segment lamellae in xenopus laevis rod and cone photoreceptors. *Investigative Ophthalmology and Visual Science* **53**, 361–373 (2012).
17. Carr, B. J., Stanar, P. & Moritz, O. L. Distinct roles for prominin-1 and photoreceptor cadherin in outer segment disc morphogenesis in CRISPR-altered X. laevis. *Journal of cell science* **134**, (2021).
18. Röhlich, P., Adams, G., McDowell, J. H. & Hargrave, P. A. Binding Pattern of Anti-rhodopsin Monoclonal Antibodies to Photoreceptor Cells: An Immunochemical Study. *Exp Eye Res* **49**, 999–1013 (1989).
19. Palinski, W. *et al.* Cloning of monoclonal autoantibodies to epitopes of oxidized lipoproteins from apolipoprotein E-deficient mice. Demonstration of epitopes of oxidized low density lipoprotein in human plasma. *J. Clin. Invest.* **98**, 800–814 (1996).
