# Supplementary material for: prominin-1-null Xenopus laevis develop subretinal drusenoid-like deposits, cone-rod dystrophy and RPE atrophy
Source: J Cell Sci. 2024 Nov 12;137(21):jcs262298. doi: 10.1242/jcs.262298 (PMC11586525; doi:10.1242/jcs.262298)
Supplement: Supplementary file 1 [file joces-137-262298_review_history.pdf]

prominin-1-null *Xenopus laevis* develop subretinal drusenoid-like deposits, cone-rod dystrophy and RPE atrophy

Brittany J. Carr, Dominic Skitsko, Linnea M. Kriese, Jun Song, Zixuan Li, Myeong Jin Ju and Orson L. Moritz
DOI: 10.1242/jcs.262298

Editor: Caroline Hill

Review timeline
Original submission: 4 June 2024
Editorial decision: 29 July 2024
First revision received: 10 September 2024
Editorial decision: 16 September 2024
Second revision received: 17 September 2024
Accepted: 19 September 2024

Original submission

First decision letter

MS ID#: JOCES/2024/262298

MS TITLE: *Prominin-1* null *Xenopus laevis* develop subretinal drusenoid-like deposits, cone-rod dystrophy, and dysmorphic retinal pigment epithelium.

AUTHORS: Brittany J Carr, Dominic Skitsko, Jun Song, Zixuan Li, Myeong Jin Ju, and Orson L Moritz
ARTICLE TYPE: Research Article

We have now reached a decision on the above manuscript.

To see the reviewers' reports and a copy of this decision letter, please go to: https://submit-jcs.biologists.org and click on the 'Manuscripts with Decisions' queue in the Author Area. (Corresponding author only has access to reviews.)

As you will see, both reviewers gave favourable reports but raised some critical points that will require amendments to your manuscript. I hope that you will be able to carry these out because I would like to be able to accept your paper, depending on further comments from reviewers. Most of the revisions can be done with textual changes, but there is one experiment suggested by Reviewer 1 that I would like you to do if possible.

Please ensure that you clearly highlight all changes made in the revised manuscript. Please avoid using 'Tracked changes' in Word files as these are lost in PDF conversion.

I should be grateful if you would also provide a point-by-point response detailing how you have dealt with the points raised by the reviewers in the 'Response to Reviewers' box. Please attend to all of the reviewers' comments. If you do not agree with any of their criticisms or suggestions please explain clearly why this is so.

Reviewer 1

Advance summary and potential significance to field

© 2024. Published by The Company of Biologists under the terms of the Creative Commons Attribution License (https://creativecommons.org/licenses/by/4.0/).

This manuscript by Carr et al. reports a novel finding that is consistent with the authors' suggestion that X. laevis can be potentially an informative experimental animal model for understanding fundamental processes involved in human retinal degenerations, including age-related macular degeneration (AMD), Stargardt's disease, etc. They convincingly demonstrate truncated mutation (deletion) of prominin-1 (PROM1) causes an age-dependent formation and accumulation in the inter-photoreceptor/subretinal space of material with features consistent with subdrusenoid deposits (SDDs), as observed in postmortem eyes from AMD patients. The manuscript is clearly written and easy to follow.

Methodologically, the study was performed meticulously-- the methods utilized were appropriate for the scope and intent of the study and the data obtained therefrom are of high quality and certainly suitable for publication. The authors provide a very informative, yet succinct, introduction to provide necessary background to the reader and to provide context regarding why the study was performed in the first place. The conclusions drawn from the study findings are reasonable, for the most part. They posit that, beyond initially (primarily) causing structural and likely functional abnormalities in the retinal photoreceptor cells, prominin-1 mutations cause secondary formation of SDDs that those deposits compromise the function and health of the adjacent retinal pigment epithelium (RPE), significantly contributing to the ensuing progressive, irreversible retinal degeneration.

Comments for the author

A few comments and questions for the authors to consider (that I believe warrant revisions):

1. The rationale for the use of CRISPR/Cas-driven truncation of prominin-1 is that prominin-1 is a gene product essential for photoreceptor outer segment disc membrane morphogenesis, and that in other (e.g., rodent) animal models prominin-1 mutation or deletion causes a progressive retinal degeneration and in humans it has been associated with progressive, irreversible diseases such as Stargardt's maculopathy. However, there's no mention that prominin-1 is also expressed in the RPE. (See Bhattacharya et al. IOVS, 2017-- which is not even cited. Those authors also reported the prominin-1 is a major regulator of authophagy in the RPE.) Hence, it is more than likely that mutation or deletion of prominin-1 could have direct cell-autonomous effects (not obligatorily dependent on defects caused directly in photoreceptor cells). Authors should acknowledge the previously published work and consider this in the context of their study conclusions and the Discussion section.

2. J Cell Sci is a broad-based biological science journal-- so the authors shouldn't assume that the readership is fully knowledgeable about the fact that the retina of X. laevis, unlike humans and nonhuman primates, lacks a macula and that compared to humans (which then begs the question- "How can this amphibian serve as a faithful "model" for AMD?"). Also that their retina is relatively "cone-rich", compared to that of humans and nonhuman primates. I suggest that the Introduction should include those facts.

3. The authors posit that the accumulation of the SDD-like material provokes oxidative stress in the RPE, which in turn contributes to retinal degeneration. They also state that the molecular composition of SDDs has not yet been fully reported, but histologically and by cytochemical staining it appears to be compositionally different from what has been reported for bona fide drusen in postmortem human ocular specimens. Have the authors performed immunohistochemical analyses that would demonstrate oxidatively modified proteins (e.g., 4-hydroxynonenal) or lipids (e.g., malondialdehyde) being present in the SDD-like material? That would be relatively easy and would bolster their hypothesis.

<u>Reviewer 2</u>

Advance summary and potential significance to field

The authors previously found that Prom1 knockout frog displayed severely dysmorphic photoreceptors, such as overgrown and disorganized disc membranes. In this manuscript, the

© 2024. Published by The Company of Biologists under the terms of the Creative Commons Attribution License (https://creativecommons.org/licenses/by/4.0/).

authors tracked the retinal degeneration in old prom1 null frogs. They found the retinal degeneration of prom-null frog are age-related and likely caused by PRE death and damage. The aged prom1-null frog retina developed SDD-like deposits accumulated in size and number, and they are present before retinal degeneration occurs. The authors think that, instead of disrupted outer segment morphogenesis, the secondary toxic effects from these SDD-like deposits or dysfunction or death of RPE may be responsible for prom1-related retinal degeneration. The conclusions are sound and provide new sight in prom1-related retinal dystrophy. The data are generally convincing.

Comments for the author

A few specific comments follow:

1. Does Prom1 expressed in RPE? In Figure S2, it is seemed that prom1 predominantly expressed in OS of photoreceptor, not in RPE.

2. In Figure 3, large deposits can't be labeled by RPE65. To verify the RPE origin of SDD-like deposits, immunoreactivity of other RPE markers may be required.

3. In figure 5, TEM data suggested MVBs are present in prom1-null RPE. Some studies indicated that Prom1 is involved in the autophagy maintenance in RPE. Are there any evidences or clues that the SDD-like deposits in prom1-null retina caused by impaired autophagy?

4. In last results sections (line 315-325), before describing the CTBP2 and calbindin immunoreactivity results, the authors should indicate the meaning of CTBP2 and calbindin immunoreactivity.

5. line 342, there are no figure 10B.

First revision

<u>Author response to reviewers' comments</u>

We thank the reviewers for their comments, and are pleased that our work was reviewed so positively. We have addressed reviewer comments as follows. Changes in the manuscript that address reviewer comments are highlighted in yellow and page numbers and lines are denoted where appropriate.

Reviewer 1 Comments for the author

1. Re: Discussion of work by Bhattacharya et al. 2017.

We are aware of the work performed by Bhattacharya et al. that reports functional PROM1 in the RPE that regulates autophagy through mTOR. We were initially interested in this finding as it could help to explain our findings. Upon further investigation, in the literature and with our own work, however, we are not convinced that fully differentiated RPE expresses functional prom1 protein. We have never seen prom1 immunofluorescence in the RPE in our own studies of fully differentiated RPE tissue (n = 64 wildtype animals surveyed at different developmental time points), and Bhattacharya et al. themselves state that prom1 protein expression is negatively correlated with RPE differentiation. Moreover, RNA sequencing data from the NEI eyeIntegration database does not support the presence of PROM1 RNA in fully differentiated RPE or RPE cell cultures, including ARPE-19, hTERT-RPE, D407 or primary cultures.

We have added the Bhattacharya references to our discussion (Page 19-20, lines 492-504) and contrasted it to our work. We have also added a representative micrograph of prom1 immunofluorescence in the frog retina including the RPE and a heat map constructed from the NEI eyeIntegration Database data (Supplementary Fig. S6).

2. Re: Discussion of frog retinal mosaic and lack of macula.

© 2024. Published by The Company of Biologists under the terms of the Creative Commons Attribution License (https://creativecommons.org/licenses/by/4.0/).

We thank the reviewer for this comment. We have addressed these points in the discussion, where we speak to the feasibility of frogs as a model of AMD-like disease. We have also briefly introduced this idea earlier in the introduction. (Page 2, lines 50-56; Page 18, 449-458).

3. Re: fluorescence labeling of oxidative stress.
We thank the reviewer for their suggestions of additional markers to label markers to search for oxidative stress. We have included immunofluorescence for E06, a marker of oxidized phospholipids. These data are included in revised figure 2 and discussed in the results (Page 11, lines 273-280).

Reviewer 2 Comments for the author

1. Re: Is PROM1 expressed in the RPE?
Please see response #1 to reviewer 1. We have added this discussion to the manuscript and added a supplementary figure (Supplementary Figure S6).

2. Re: Lack of RPE65 labeling in the large deposits.
We believe that there is no RPE65 labeling in the larger deposits because the protein would have been broken down to a degree that antibody labeling is not longer effective. We agree, however, that this is not the only explanation, as the deposits do label positively for other markers, such as vimentin. We have tried to identify more RPE markers to test, but to date, none have been successful due to lack of cross-reactivity with frog (i.e., they do not label wildtype RPE either). For now, we depend on indirect indicators that these could be of RPE origin such as breakdown of RPE (Fig. 2B, 4C,E,G,I, Supplementary Fig. S2), pigment granule infiltration into the deposits (Fig. 2B,F), red autofluorescence (Fig. 2C,D) and now, E06 labelling patterns (Fig. 2 E,F), which show infiltration of autofluorescence and oxidized phospholipids into the OS layer, likely from the RPE as wildtype have similar, but reduced labeling patterns.

3. Re: Are there any clues that SDD-like deposits are caused by impaired autophagy?
Figures 1 and 5 show build-up of waste products in prom1-null RPE compared to wildtype animals. This could be due to impaired autophagy, but it is not the only explanation. We hope to study this further in the future, but have been hindered by the establishment of a new lab and re-derivation of prom1-null frog lines. As such, we do not currently have aged frog tissues to study this. Thus, we consider this question a topic of future investigation beyond the scope of the present manuscript, which aims to describe a phenotype in a new animal model.

4. Re: Defining CTBP2 and meaning of CTBP2 and calbindin labeling. Addressing missing figure.
We thank the reviewer for catching these errors. We have corrected them and added context for the purposes of CTBP2 and calbindin labelling. Page 14, lines 347-351.

<u>Second decision letter</u>

MS ID#: JOCES/2024/262298

MS TITLE: *Prominin-1* null *Xenopus laevis* develop subretinal drusenoid-like deposits, cone-rod dystrophy, and RPE atrophy

AUTHORS: Brittany J Carr, Dominic Skitsko, Jun Song, Linnea M Kriese, Zixuan Li, Myeong Jin Ju, and Orson L Moritz
ARTICLE TYPE: Research Article

We have now reached a decision on the above manuscript.

I have gone through your revisions and I feel that you have adequately addressed the reviewers' comments and therefore consider that the paper is now suitable for publication in J. Cell Sci.

© 2024. Published by The Company of Biologists under the terms of the Creative Commons Attribution License (https://creativecommons.org/licenses/by/4.0/).

However, I noticed that for some of the figures (Figure 1, Figure 2 and Figure 7) the molecules that were being stained for were not labelled in the figure. It would be much clearer if you could do this, as you have for Figure 3 for instance.

Once this is done and you haveresubmitted the manuscript, I will be happy to accept it.

Second revision

Author response to reviewers' comments

We thank the reviewers for their comments, and are pleased that our work was reviewed so positively. We have addressed reviewer comments as follows. Changes in the manuscript that address reviewer comments are highlighted in yellow and page numbers and lines are denoted where appropriate.

Reviewer 1 Comments for the author

1. Re: Discussion of work by Bhattacharya et al. 2017.
We are aware of the work performed by Bhattacharya et al. that reports functional PROM1 in the RPE that regulates autophagy through mTOR. We were initially interested in this finding as it could help to explain our findings. Upon further investigation, in the literature and with our own work, however, we are not convinced that fully differentiated RPE expresses functional prom1 protein. We have never seen prom1 immunofluorescence in the RPE in our own studies of fully differentiated RPE tissue (n = 64 wildtype animals surveyed at different developmental time points), and Bhattacharya et al. themselves state that prom1 protein expression is negatively correlated with RPE differentiation. Moreover, RNA sequencing data from the NEI eyeIntegration database does not support the presence of PROM1 RNA in fully differentiated RPE or RPE cell cultures, including ARPE-19, hTERT-RPE, D407 or primary cultures.

We have added the Bhattacharya references to our discussion (Page 19-20, lines 492-504) and contrasted it to our work. We have also added a representative micrograph of prom1 immunofluorescence in the frog retina including the RPE and a heat map constructed from the NEI eyeIntegration Database data (Supplementary Fig. S6).

2. Re: Discussion of frog retinal mosaic and lack of macula.
We thank the reviewer for this comment. We have addressed these points in the discussion, where we speak to the feasibility of frogs as a model of AMD-like disease. We have also briefly introduced this idea earlier in the introduction. (Page 2, lines 50-56; Page 18, 449-458).

3. Re: fluorescence labeling of oxidative stress.
We thank the reviewer for their suggestions of additional markers to label markers to search for oxidative stress. We have included immunofluorescence for E06, a marker of oxidized phospholipids. These data are included in revised figure 2 and discussed in the results (Page 11, lines 273-280).

Reviewer 2 Comments for the author

1. Re: Is PROM1 expressed in the RPE?
Please see response #1 to reviewer 1. We have added this discussion to the manuscript and added a supplementary figure (Supplementary Figure S6).

2. Re: Lack of RPE65 labeling in the large deposits.
We believe that there is no RPE65 labeling in the larger deposits because the protein would have been broken down to a degree that antibody labeling is not longer effective. We agree, however,

© 2024. Published by The Company of Biologists under the terms of the Creative Commons Attribution License (https://creativecommons.org/licenses/by/4.0/).

that this is not the only explanation, as the deposits do label positively for other markers, such as vimentin. We have tried to identify more RPE markers to test, but to date, none have been successful due to lack of cross-reactivity with frog (i.e., they do not label wildtype RPE either). For now, we depend on indirect indicators that these could be of RPE origin such as breakdown of RPE (Fig. 2B, 4C,E,G,I, Supplementary Fig. S2), pigment granule infiltration into the deposits (Fig. 2B,F), red autofluorescence (Fig. 2C,D) and now, E06 labelling patterns (Fig. 2 E,F), which show infiltration of autofluorescence and oxidized phospholipids into the OS layer, likely from the RPE as wildtype have similar, but reduced labeling patterns.

3. Re: Are there any clues that SDD-like deposits are caused by impaired autophagy?
Figures 1 and 5 show build-up of waste products in prom1-null RPE compared to wildtype animals. This could be due to impaired autophagy, but it is not the only explanation. We hope to study this further in the future, but have been hindered by the establishment of a new lab and re-derivation of prom1-null frog lines. As such, we do not currently have aged frog tissues to study this. Thus, we consider this question a topic of future investigation beyond the scope of the present manuscript, which aims to describe a phenotype in a new animal model.

4. Re: Defining CTBP2 and meaning of CTBP2 and calbindin labeling. Addressing missing figure.
We thank the reviewer for catching these errors. We have corrected them and added context for the purposes of CTBP2 and calbindin labelling. Page 14, lines 347-351.

Third decision letter

MS ID#: JOCES/2024/262298

MS TITLE: *Prominin-1* null *Xenopus laevis* develop subretinal drusenoid-like deposits, cone-rod dystrophy, and RPE atrophy

AUTHORS: Brittany J Carr, Dominic Skitsko, Jun Song, Linnea M Kriese, Zixuan Li, Myeong Jin Ju, and Orson L Moritz
ARTICLE TYPE: Research Article

I am happy to tell you that your manuscript has been accepted for publication in Journal of Cell Science, pending standard publication integrity checks.

© 2024. Published by The Company of Biologists under the terms of the Creative Commons Attribution License (https://creativecommons.org/licenses/by/4.0/).
